# Mild Traumatic Brain Injury Produces Neuron Loss That Can Be Rescued by Modulating Microglial Activation Using a CB2 Receptor Inverse Agonist

**DOI:** 10.3389/fnins.2016.00449

**Published:** 2016-10-06

**Authors:** Wei Bu, Huiling Ren, Yunping Deng, Nobel Del Mar, Natalie M. Guley, Bob M. Moore, Marcia G. Honig, Anton Reiner

**Affiliations:** ^1^Department of Anatomy and Neurobiology, University of Tennessee Health Science CenterMemphis, TN, USA; ^2^Department of Pharmaceutical Sciences, University of Tennessee Health Science CenterMemphis, TN, USA; ^3^Department of Ophthalmology, University of Tennessee Health Science CenterMemphis, TN, USA

**Keywords:** traumatic brain injury, neuron loss, neuron rescue, cerebral cortex, striatum, basolateral amygdala, cannabinoid type-2 receptor inverse agonist

## Abstract

We have previously reported that mild TBI created by focal left-side cranial blast in mice produces widespread axonal injury, microglial activation, and a variety of functional deficits. We have also shown that these functional deficits are reduced by targeting microglia through their cannabinoid type-2 (CB2) receptors using 2-week daily administration of the CB2 inverse agonist SMM-189. CB2 inverse agonists stabilize the G-protein coupled CB2 receptor in an inactive conformation, leading to increased phosphorylation and nuclear translocation of the cAMP response element binding protein (CREB), and thus bias activated microglia from a pro-inflammatory M1 to a pro-healing M2 state. In the present study, we showed that SMM-189 boosts nuclear pCREB levels in microglia in several brain regions by 3 days after TBI, by using pCREB/CD68 double immunofluorescent labeling. Next, to better understand the basis of motor deficits and increased fearfulness after TBI, we used unbiased stereological methods to characterize neuronal loss in cortex, striatum, and basolateral amygdala (BLA) and assessed how neuronal loss was affected by SMM-189 treatment. Our stereological neuron counts revealed a 20% reduction in cortical and 30% reduction in striatal neurons bilaterally at 2–3 months post blast, with SMM-189 yielding about 50% rescue. Loss of BLA neurons was restricted to the blast side, with 33% of Thy1+ fear-suppressing pyramidal neurons and 47% of fear-suppressing parvalbuminergic (PARV) interneurons lost, and Thy1-negative fear-promoting pyramidal neurons not significantly affected. SMM-189 yielded 50–60% rescue of Thy1+ and PARV neuron loss in BLA. Thus, fearfulness after mild TBI may result from the loss of fear-suppressing neuron types in BLA, and SMM-189 may reduce fearfulness by their rescue. Overall, our findings indicate that SMM-189 rescues damaged neurons and thereby alleviates functional deficits resulting from TBI, apparently by selectively modulating microglia to the beneficial M2 state. CB2 inverse agonists thus represent a promising therapeutic approach for mitigating neuroinflammation and neurodegeneration.

## Introduction

Mild traumatic brain injury (TBI) occurs frequently as an outcome from military, sports, and recreational activities, and vehicular accidents, and can lead to a variety of adverse sensory, motor, cognitive and emotional outcomes (Faul et al., 2010; Risdall and Menon, 2011; Johnson et al., 2013). Mild TBI involves either brief or no loss of consciousness and causes minimal overt brain destruction, but produces widespread axonal injury that is commonly referred to as “diffuse” axonal injury, and sets in motion subsequent secondary degenerative events (Bazarian et al., 2013; Johnson et al., 2013; Smith et al., 2013b). The initial injury appears to stem from the compressive, tensile, and shear forces exerted on neural tissue by the concussive force created by a blow to the head with a blunt object, a blast shock wave, or a rapid head acceleration—deceleration (Namjoshi et al., 2013). Although a variety of treatments have been tested in animal models and human clinical trials (Xiong et al., 2013), effective therapies have not been developed. Since microglial activation is one of the secondary events that appears to worsen the outcome after mild TBI (Kelley et al., 2007; Redell and Dash, 2007; Cao et al., 2012; Das et al., 2012; Kumar and Loane, 2012; Patterson and Holahan, 2012; Perez-Polo et al., 2013; Smith et al., 2013a), we have evaluated the benefits of a novel pharmacological approach for reducing the harmful effects of activated microglia (Reiner et al., 2015).

We created mild TBI in mice using a focal blast model (Heldt et al., 2014; Guley et al., 2016). As our pharmacological approach, we modulated activated microglia via their cannabinoid type 2 receptors (CB2) (Schomberg and Olson, 2012; Cherry et al., 2014). CB2 receptors are typically expressed at very low levels in brain, are more concentrated in microglia than neurons, and are thus non-psychotropic, in contrast to cannabinoid type 1 (CB1) receptors (Benito et al., 2003; Stella, 2010; Baek et al., 2013). Activated microglia, however, rapidly increase their expression of CB2 receptors (Benito et al., 2003; Ashton and Glass, 2007; Stella, 2010; Schomberg and Olson, 2012; Baek et al., 2013; Donat et al., 2014), and so drugs acting on CB2 are especially promising for selectively targeting microglia for therapeutic purposes. CB2 inverse agonists in particular represent a unique class of ligands with promise for beneficially modulating microglia to treat TBI (Lunn et al., 2006, 2008). CB2 inverse agonists stabilize CB2 receptors in an inactive state, which are otherwise constitutively active, and reduce adenylyl cyclase inhibition and thereby increase cAMP production (Atwood et al., 2012). This in turn leads to downstream activation of protein kinase A, which phosphorylates the cAMP response element binding protein (CREB). Increased phosphorylation and nuclear translocation of CREB appear to bias activated microglia from the M1 toward the M2 state, and thus underlie the anti-inflammatory and pro-repair effects of CB2 inverse agonists (Lunn et al., 2006, 2008; Lawrence and Natoli, 2011; Presley et al., 2015).

We have previously tested if the selective CB2 inverse agonist SMM-189, which was developed in one of our laboratories and has striking efficacy in reducing the M1 features and increasing the M2 features of human and murine microglia *in vitro* (Presley et al., 2015; Reiner et al., 2015), could improve the outcome after mild TBI in our mouse model. We have shown that a 2-week daily treatment with SMM-189 after mild TBI greatly attenuates the motor deficits, visual deficits and increased fearfulness that are otherwise evident 2–6 weeks after the traumatic event (Reiner et al., 2015). We have also found that the decrease in some of these functional abnormalities was associated with reduced pathology in neural structures associated with these functions. For example, rescue of visual deficits was associated with reduced thinning of the retina, and the attenuation in fearfulness was associated with the rescue of at least one population of neurons in the basolateral amygdala (BLA) that promotes fear. Our previous work has thus shown that treatment with a CB2 receptor inverse agonist that biases brain microglia from a pro-inflammatory M1 phenotype to a pro-healing M2 phenotype is beneficial after mild TBI (Reiner et al., 2015).

The present study had two goals. First, we sought to determine if mild TBI resulted in the loss of neurons in brain regions linked to motor functions and fear control, to better understand the basis of the motor and emotional deficits observed in our model. To this end, we used blinded stereology in mice 2–3 months after mild TBI to analyze neuron loss in cerebral cortex and striatum because of their role in motor control, and several neuron types in the BLA that are differentially involved in fear regulation and expression. Secondly, we evaluated the efficacy of SMM-189 in rescuing these neuron types, both to better understand the basis of the functional recovery SMM-189 provides, and also to further assess the contributions of the neuronal loss to the functional deficits. As part of this, we confirmed that SMM-189 treatment increased levels of nuclear pCREB in microglia in the brain systems under study, which would thereby bias the microglia toward an M2 state. Overall, our findings indicate diffuse neuron loss as a contributor to functional deficits in our TBI model, and they support the use of CB2 inverse agonists as an approach for reducing neuron loss and/or injury after mild TBI and attenuating functional deficits.

## Materials and methods

### Animals

Three-month old male mice were subjected to single left-side blasts of 0-psi (sham) or 50–60-psi above atmospheric pressure and the outcome evaluated in two sets of studies. In one set of studies, we assessed if SMM-189 treatment increases nuclear pCREB levels in microglia, using pCREB/CD68 double immunofluorescent labeling of brain sections from mice 3 days either after blast alone or after blast followed by SMM-189 treatment. In a second set of studies, we evaluated the TBI outcome for neuron abundance in cerebral cortex, striatum and BLA histologically 2–3 months after blast, to allow time for any neuron loss to develop, comparing mice with and without SMM-189 treatment. Two strains of mice were used, C57BL/6 mice and reporter mice (on a C57BL/6 background) conditionally expressing enhanced yellow fluorescent protein (EYFP) in Thy1-expressing telencephalic neurons of the *emx1* lineage (Gorski et al., 2002; Bareyre et al., 2005). The EYFP reporter mice were used to histologically evaluate the effects of blast on the Thy1+ vs. Thy1−negative subset of functionally distinct excitatory pyramidal neurons of BLA (Heldt et al., 2014; Reiner et al., 2015). The C57BL/6 mice were either purchased from Jackson Laboratories (JAX; Bar Harbor, ME), and/or taken from a colony maintained from C57BL/6 founders from JAX at the University of Tennessee Health Science Center (UTHSC). Floxed Thy1-EYFP reporter mice (purchased from JAX) and *emx1*-Cre driver mice (purchased from the Mutant Mouse Regional Resource Consortium) were maintained as colonies at the UTHSC, and bred to one another to produce the Thy1-EYFP+/emx1-cre+ progeny used for the experiments. Mice were injected intraperitoneally daily with 6 mg/kg SMM-189 or vehicle (ethanol:Cremophor:0.9% saline; 5:5:90) for 14 days beginning 2 h after 50–60 psi or 0-psi (sham) blast in the case of neuronal loss studies and for 3 days in the case of pCREB studies. The dose used was chosen based on studies of uptake in rodent brain of structurally similar tri-aryl CB2 compounds (Fujinaga et al., 2010), which proved effective in our prior study (Reiner et al., 2015), and which is more than adequate for CB2 receptor activation given its 121.3 nM affinity (Presley et al., 2015). Note that although Cremophor has been linked to neuropathy when used as a vehicle for anti-cancer drugs (Gelderblom et al., 2001), the dose of Cremophor used here was below the maximum recommended dose for rodents, which is not known to have side effects (Neervannan, 2006). Consistent with this, no abnormalities were seen in optic nerve of mice following 2 weeks of Chremophor vehicle treatment in a prior study by us (Reiner et al., 2015). In addition, the number of cortical and striatal neurons in mice that received sham blast and Cremophor vehicle in the present study did not differ from those in our prior study (Guley et al., 2016) that received sham blast and no vehicle, and there were also no differences in body weight. All studies were performed in accordance with an UTHSC Institutional Animal Care and Use Committee approved protocol and complied with the National Institutes of Health and Society for Neuroscience guidelines. The mice analyzed here for neuron loss included some that had been used in our initial study of functional and morphological aspects of SMM-189 benefit (16; Reiner et al., 2015). For studies of neuron loss in cortex and striatum, 3 mice were analyzed per group (0-psi vehicle, 50-psi vehicle, and 50-psi, SMM-189), with a mean post-blast survival of 75.3, 75.7, and 77.0 days, respectively, per group. For BLA neuron loss, 4 mice were analyzed per group, with a mean post-blast survival of 69.3, 67.5, and 58.0 days, respectively, per group.

### TBI methods

The overpressure air blast was delivered by a small horizontally mounted air cannon system (Heldt et al., 2014; Guley et al., 2016), consisting of a modified paintball gun (Invert Mini, Empire Paintball, Sewell NJ), pressurized air tank, and x-y table secured onto a medium-density fiberboard. The original paintball gun barrel with a 13 mm aperture was replaced with a machined barrel with a 6.5 mm diameter aperture, to increase output pressure. The air blast pressures from the paintball gun were controlled by adjusting the output from a pressurized air tank, as monitored by a gun input pressure gauge. The part of the mouse exposed to the blast was restricted to a 7.5 mm diameter mid-cranial territory, as described previously (Guley et al., 2016). The exposed region was the parietal area of the left side of the head between the ear and the eye, encompassing that part of the skull overlying the forebrain. A foam rubber sleeve surrounding the mouse cushioned the non-blast side of the mouse, to stabilize it and minimize head displacement. Prior to blast exposure, mice were anesthetized with avertin (400 mg/kg body weight), the fur of the parietal region of the left side of the head shaved, and the mouse secured in a holder as described previously (Guley et al., 2016). TBI mice used for study of cortex and striatum received 50-psi blast, while TBI mice used for study of BLA received 60-psi blast. All of the same procedures were followed for sham-blast mice (0-psi), except that the mouse was shielded from the blast by a metal plate inserted between it and the gun nozzle. Mice received 35 mg/ml Tylenol in their drinking water for 24 h after blast.

### Morphological methods

Histological analysis was carried out on fixed brain tissue to determine the effects of TBI on the brain, and the remedial effects of SMM-189. Either 3 days or 2–3 months after blast, mice were deeply anesthetized (avertin; 0.2 ml/g body weight), the chest opened, and 0.1 ml of heparinized saline (800 U.S.P. units/ml) injected into the heart. Mice were then perfused transcardially with 40 ml of 0.9% NaCl in 0.1 M sodium phosphate buffer at pH 7.4 (PB), followed by 200 ml of 4% paraformaldehyde, 0.1 M lysine-0.1 M sodium periodate in 0.1 M PB at pH 7.4 (PLP). The brain was removed, post-fixed overnight, a pin inserted longitudinally into the right side to distinguish left blast-exposed side from right non-exposed side, and then stored in a 20% sucrose/10% glycerol solution at 4°C. The fixed brains were sectioned frozen on a sliding microtome in the transverse plane at 35 μm, and each brain collected as either 6 (for BLA) or 12 separate series (for cortex and striatum) in 0.1 M PB with 0.02% sodium azide. At least one series of brain sections from each mouse was mounted as sectioned and stained with cresyl violet. One or more series were immunostained for the pan-neuronal marker NeuN using a mouse monoclonal antibody (Millipore Corp., Billerica, MA) and peroxidase-antiperoxidase (PAP) procedures as described previously (Reiner et al., 2015; Guley et al., 2016), and in the case of 2–3 month survival mice employed to count all neuronal perikarya in cerebral cortex, striatum and BLA. Thy1+ pyramidal neurons of BLA in the Thy1 reporter mice were visualized for transmitted light microscopic study using a mouse monoclonal antibody against green fluorescent protein (Rockland Inc, Gilbertsville, PA), which crossreacts with EYFP due to the high similarity of their antigenic determinants. We detected PARV+ interneurons in BLA using a mouse monoclonal anti-PARV antibody (Sigma-Aldrich, St. Louis, MO), as described previously (Deng et al., 2007). Thy1+ pyramidal neurons and PARV+ interneurons in BLA were each visualized using PAP procedures in separate series of coronal sections. To evaluate CREB phosphorylation by SMM-189, we used a rabbit anti-pCREB antibody (#p1010-133; PhosphoSolutions, Aurora, CO) and anti-rabbit IgG conjugated to Alexa-594 (Molecular Probes; Eugene, OR), in conjunction with a rat anti-CD68 antibody (#ab53444; Abcam, Cambridge, MA) and anti-rat IgG conjugated to Alexa-647 (Molecular Probes; Eugene, OR) to identify microglia. Nuclei were visualized by DAPI staining. Sections were mounted on gelatin-coated slides and coverslipped with ProLong® antifade medium (Molecular Probes, Eugene, OR). Sections were viewed and images captured using a Zeiss 710 confocal laser-scanning microscope.

### Neuron counts

Unbiased, blinded stereological neuron counts were carried out using Stereo Investigator (Micro-Brightfield, Colchester, VT) with the optical fractionator method, as described previously (Reiner et al., 2012; Guley et al., 2016). Stereology also provided information on the volumes of the structures examined. The results for different groups of mice were compared using ANOVA followed by planned comparisons with post-hoc Bonferroni tests. No inhomogeneity of variances was detected by the Levene statistic for any of the data sets. Neuron counts for cortex and striatum were obtained using a one-in-twelve series of coronal sections immunolabeled for NeuN from C57BL/6 mice that had previously been used in rotarod assessments of motor performance (Reiner et al., 2015). The cortical field counted extended from the pial surface to the external capsule, and from the midline to the rhinal fissure. The striatum was defined by the contours of the external capsule and globus pallidus, and included nucleus accumbens. Cortex and striatum counts were performed from the rostral end of each to the level of the passage of the internal capsule into the thalamus, and thus included all but the most caudal ~25% of cortex and the most caudal ~10% of striatum. We have interpreted a reduction in the number of immunolabeled neurons at 2–3 months post-blast to represent neuron loss, as is routinely the case (Kordower et al., 2001; Shi et al., 2004; Unal-Cevik et al., 2004; Avramescu et al., 2009; Reiner et al., 2012; Zhang et al., 2015), although the possibility exists that the loss of immunolabeling could reflect neuronal dysfunction. To evaluate the impact of neuron loss/rescue on motor function, we used regression analysis to determine if neuron abundance was correlated with rotarod performance, as determined in the previously reported functional assessment at 2 weeks post-blast. Neuron counts for BLA were obtained from Thy1 reporter mice, which had not been tested for fearfulness, using separate one-in-six series of brain sections immunolabeled for NeuN, Thy1 (i.e. GFP) or PARV, and covered the full extent of rostral BLA.

## Results

### Activation of CREB by SMM-189

**Figure 1:**
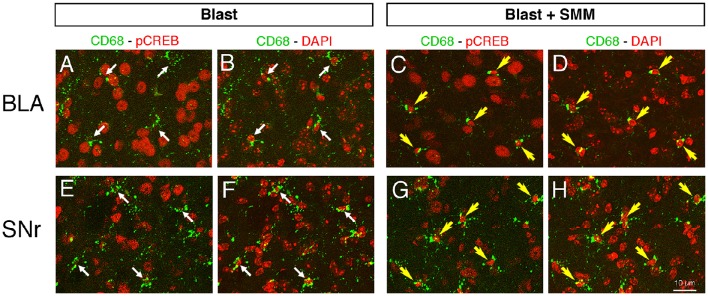
**Images showing the effect of SMM-189 treatment on pCREB expression in microglia after blast**. Confocal image pairs, one showing immunostaining for pCREB and CD68 and the other showing pCREB and DAPI, in left BLA and SNr in a blast alone mouse **(A,B,E,F)** compared to a SMM-189 treated blast mouse **(C,D,G,H)** at 3 days post blast. The CD68/pCREB double labeling shows microglia with CREB activation (i.e., nuclear pCREB; yellow arrows). The DAPI staining (pseudo-colored red for ease of viewing) reveals nuclei. Note that the nuclei of neurons are distinctly larger than those of microglia. The nuclei of all microglia in the blast mouse lack prominent pCREB (thin white arrows), whereas in the SMM-189 treated blast mouse all microglia are rich in nuclear pCREB (yellow arrows). Thus, SMM-189 treatment after blast results in CREB activation in microglia in BLA and SNr.

**Figure 2:**
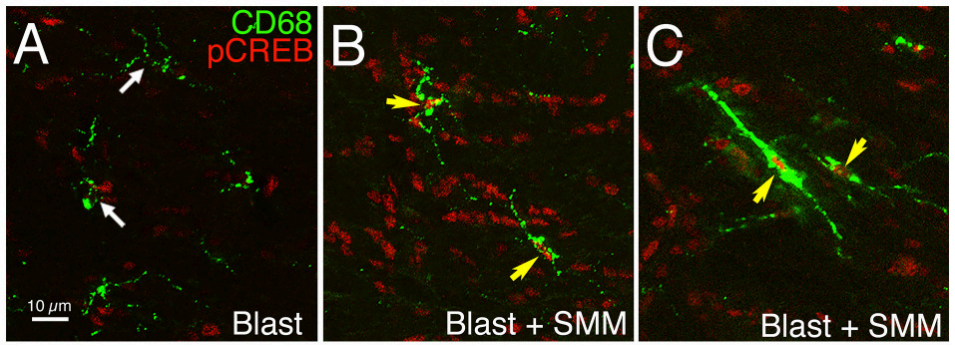
**Images showing the effect of SMM-189 treatment on pCREB expression in microglia in the left internal capsule after blast**. Confocal images showing immunostaining for pCREB and CD68 in a blast alone mouse **(A)** compared to a SMM-189-treated blast mouse **(B,C)** at 3 days post blast. DAPI staining (not shown) was used to identify nuclei. The nuclei of all microglia lack prominent pCREB in the blast mouse (white arrows), but all are rich in pCREB in the SMM-189-treated mouse (yellow arrows). Note that some microglia exhibit prominent CD68 expression and a rod-like morphology **(C)** after SMM-189 treatment, and some of these are aligned parallel to axons descending from the cerebral cortex.

As an inverse agonist, SMM-189 biases the CB2 receptor conformation to an inactive state (Presley et al., 2015), leading to phosphorylation of CREB, which, in turn, promotes transcription of M2 genes and represses transcription of M1 genes (Atwood et al., 2012). To examine the effects of SMM-189 on microglia in the brain, we immunolabeled for pCREB in conjunction with CD68 to visualize microglia. Confocal images from blast-alone mice 3 days after blast revealed no evident pCREB in the vast majority of microglia (>95%), with microglial cell bodies and nuclei confirmed as CD68+ and DAPI+. This was true for all brain regions examined, notably the BLA, the striatum and its major projection target, the substantia nigra pars reticulata (SNr), and for the axonal outflow of cerebral cortex in the internal capsule, as shown in Figures 1, 2. By contrast, in mice 3 days after blast with daily SMM-189, most, if not all, microglia in these brain regions were enriched in nuclear pCREB. Moreover, some microglia, notably in the internal capsule, had a rod-like morphology (Figure 2C). Some of the rod-like microglia were aligned parallel to axons in the internal capsule and some seemingly parallel to blood vessels. These rod-like microglia are reminiscent of microglia that become aligned along scratches in the tissue culture substratum, which are thought to correspond to an M2 state (Tam and Ma, 2014). Thus, SMM-189 acted on brain microglia as expected and consistent with its actions on microglia *in vitro* (Presley et al., 2015; Reiner et al., 2015).

### Cortical and striatal neuron loss after mild TBI is rescued by SMM-189

**Figure 3:**
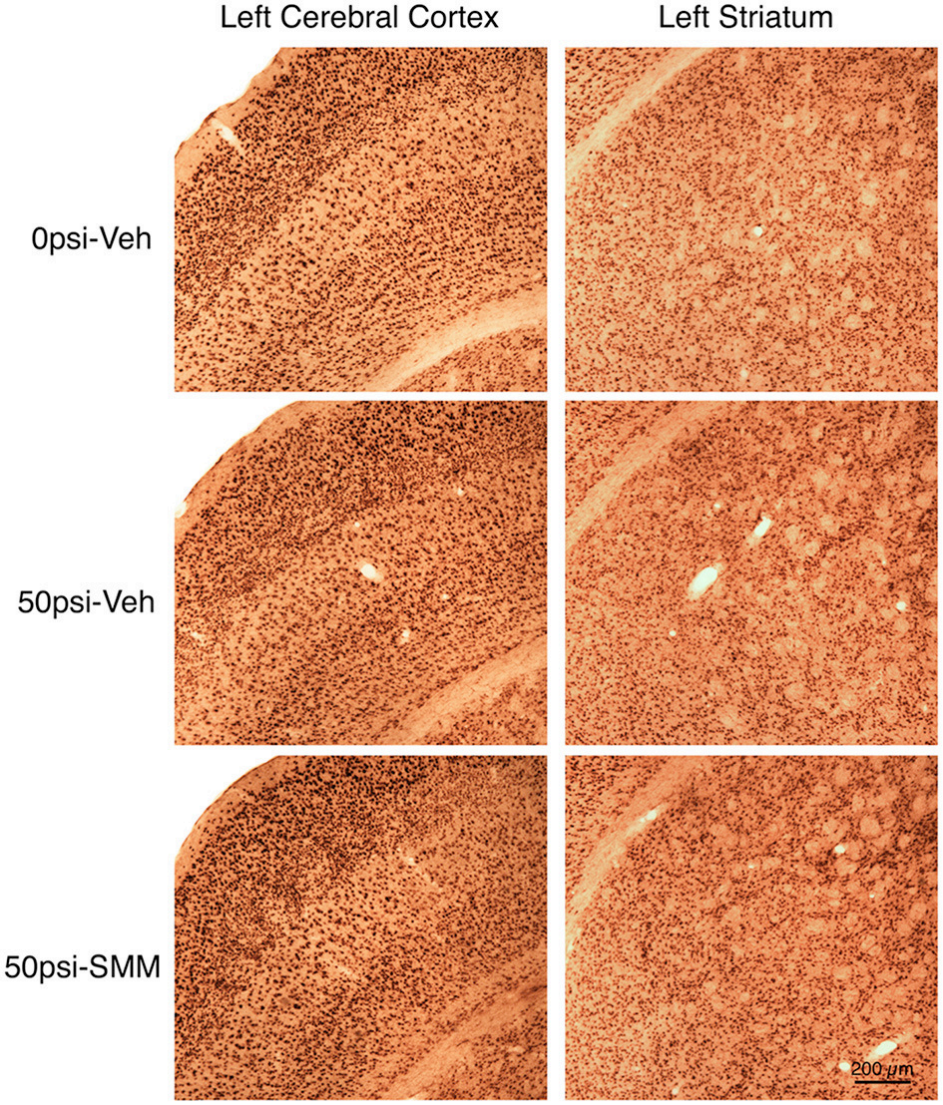
**Images showing the effect of TBI and SMM-189 on NeuN+ neurons in left cerebral cortex and striatum at 2–3 months post-blast**. Neither focal nor diffuse neuron loss is evident in the 50-psi vehicle-treated cortex or striatum, compared to 0-psi vehicle-treated cortex and striatum. Similarly, the abundance of cortical and striatal neurons does not appear obviously different in the 50-psi SMM-189 treated mice from that in either of the other two groups. Magnification is the same for all panels. Stereology, nonetheless, revealed neuron loss in both in 50-psi vehicle-treated mice.

**Figure 4:**
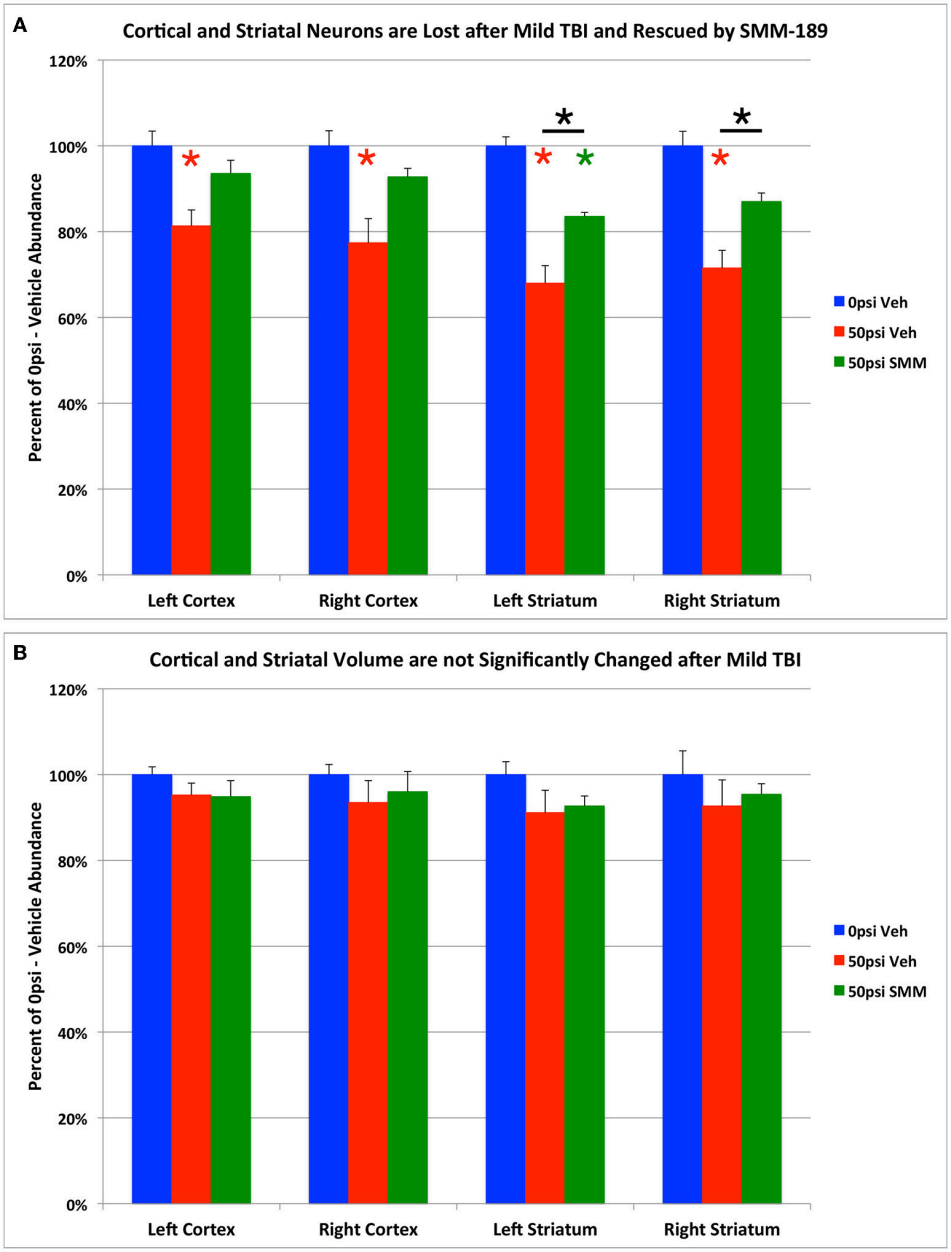
**Graphs showing the effect of TBI and SMM-189 on NeuN+ neurons (A) and on volumes (B) in cerebral cortex and striatum**. Stereological counts **(A)** revealed bilateral neuron loss in the 50-psi vehicle-treated cortex and striatum, and significant rescue after SMM-189 treatment. Red asterisks indicate a significant difference between vehicle-treated 50-psi mice and vehicle-treated 0-psi mice. Green asterisks indicate a significant difference between SMM-189 and vehicle-treated 0-psi mice. Asterisks above bars spanning the SMM-189 and vehicle-treated 50-psi mice columns indicate a significant difference between these two conditions. No significant volume loss is evident in the 50-psi vehicle-treated cortex or striatum **(B)**, compared to 0-psi vehicle-treated cortex and striatum, although a trend toward reduction is seen.

Examination of cresyl violet or NeuN stained sections did not reveal any obvious foci of neuronal loss or generalized neuron loss in cerebral cortex or striatum on either side of the brain 2–3 months after a 50-psi blast (Figure 3). However, blinded stereological neuron counts showed a bilateral loss of about 20% of neurons in the cerebral cortex (left: *p* = 0.025; right: *p* = 0.021), and a bilateral loss of about 30% of striatal neurons (left: *p* = 0.00048; right: *p* = 0.003) in mice that had received 50-psi blasts compared to those that had received 0-psi blasts (Figure 4A). The overall volumes of the cerebral cortex and the striatum were slightly, but not significantly, less in mice with 50-psi blast than in sham mice (Figure 4B). Daily treatment with SMM-189 for the 2 weeks after blast significantly reduced the cortical and striatal neuron loss, by about 50% in both cases (Figure 4A). For cortex, neuron counts in SMM-189 treated mice with 50-psi blast were no longer significantly different than in the vehicle-treated sham blast mice (left: *p* = 0.691; right: *p* = 0.745), and trended toward being more than in vehicle-treated 50-psi mice (left: *p* = 0.133; right: *p* = 0.103). For left striatum, neuron counts in SMM-189 treated mice with 50-psi blast were significantly more than in vehicle-treated 50-psi mice (p = 0.020), although they remained significantly less than in the vehicle-treated sham blast mice (*p* = 0.015). For right striatum as well, neuron counts in SMM-189 treated mice with 50-psi blast were significantly more than in vehicle-treated 50-psi mice (*p* = 0.047), but in this case they were also statistically indistinguishable from that in the vehicle-treated sham blast mice (*p* = 0.094).

**Figure 5:**
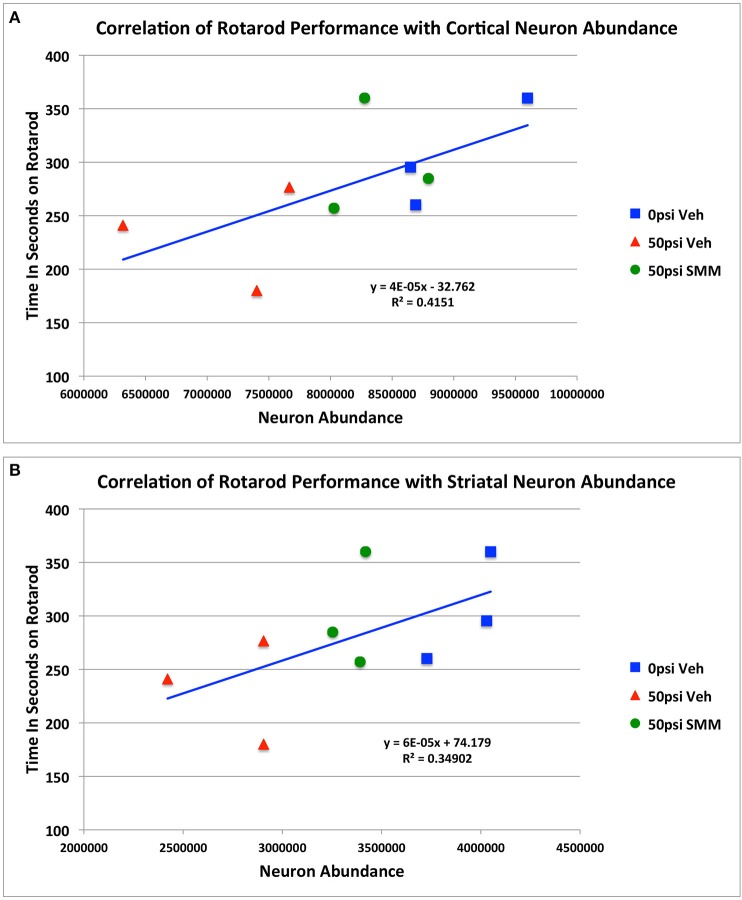
**Regression analysis showing the relationship between rotarod performance and the extent of cortical (A) and striatal (B) neuronal loss and rescue, combining the data for all three groups of mice**.

We previously reported that blast resulted in impaired rotarod performance and that SMM-189 treatment produced partial rescue of this motor deficit (Reiner et al., 2015). We used regression analysis to examine the relationship between rotarod performance and the extent of neuronal loss and rescue, combining the data for all three groups of mice (i.e., sham mice, and the TBI mice, with and without SMM-189), as shown in Figure 5. We found that cortical neuron abundance for the two sides of the brain taken together was highly correlated with rotarod performance (*r* = 0.6443; *p* = 0.032). Similarly, striatal neuron abundance for both sides of the brain combined was also highly correlated with rotarod performance (*r* = 0.5908; *p* = 0.047). These results are consistent with the idea that the loss of cortical and striatal neurons contributed to the rotarod deficit produced by blast, and that their partial rescue by SMM-189 contributed to the partial rescue of rotarod performance.

### BLA neuron loss after TBI is rescued by SMM-189

BLA is composed primarily of Thy1+ and Thy1-negative pyramidal neurons, with several types of interneurons making up the remaining 10% of the total neuronal population. The Thy1+ pyramidal neurons and the PARV+ interneurons are both reported to play a role in suppressing fear (Myers et al., 2006; Herry et al., 2008; Heldt et al., 2012; Jasnow et al., 2013). For this reason, and because we had previously observed that mild TBI results in a loss of Thy1+ neurons (Reiner et al., 2015), in the present study we used stereological neuron counting methods to more fully analyze the effects of TBI and SMM-189 treatment on neuron-type specific pattern of loss in BLA. As a first step, we determined the relative abundance of several of the different major neuron types in BLA. Using a Thy1-EYFP reporter mouse to visualize the Thy1+ neurons, and immunostaining a separate series of sections for NeuN to visualize the total neuronal population, we found that 38.1% of BLA neurons are Thy1+ neurons. As pyramidal neurons are thought to make up about 90% of the total population of BLA neurons (Godavarthi et al., 2014; Wolff et al., 2014), this suggests that Thy1-poor pyramidal neurons constitute 51.9% of all BLA neurons (90–38.1%). We also found that 3.7% of all BLA neurons are PARV+. With interneurons representing about 10% of all BLA interneurons, 6.3% of BLA neurons must correspond to the separate interneuron types that contain somatostatin, cholecystokinin, or vasoactive intestinal polypeptide (Rainnie et al., 2006; Muller et al., 2007).

**Figure 6:**
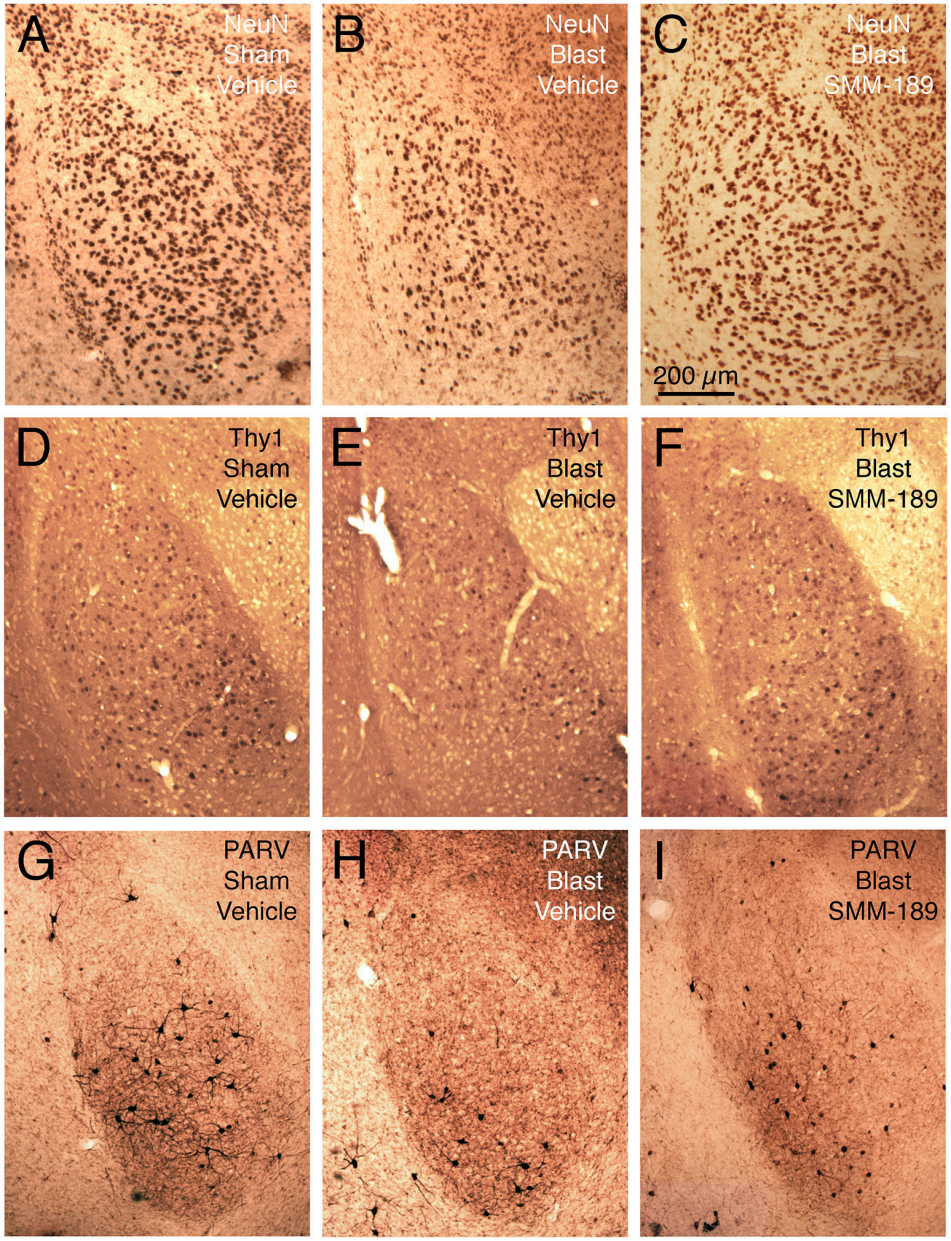
**Images showing the effect of TBI and SMM-189 on NeuN+ neurons in left BLA (A–C), on Thy1+ fear-suppressing pyramidal neurons in left BLA in a Thy1-EYFP reporter mouse (D–F), and on PARV+ interneurons in left BLA (G–I)**. A reduction in NeuN+ neurons is seen in the 60-psi blast mice (**B** compared to **A**), which trended toward being rescued by SMM-189 treatment **(C)**. Similarly, Thy1+ neurons of left BLA are reduced in the 60-psi blast mice **(E)**, and rescued in the SMM-189 treated 60-psi blast mice **(F)**, as also true for the PARV+ neurons of left BLA **(G–I)**. Medial is to the right and magnification is the same in all images.

**Figure 7:**
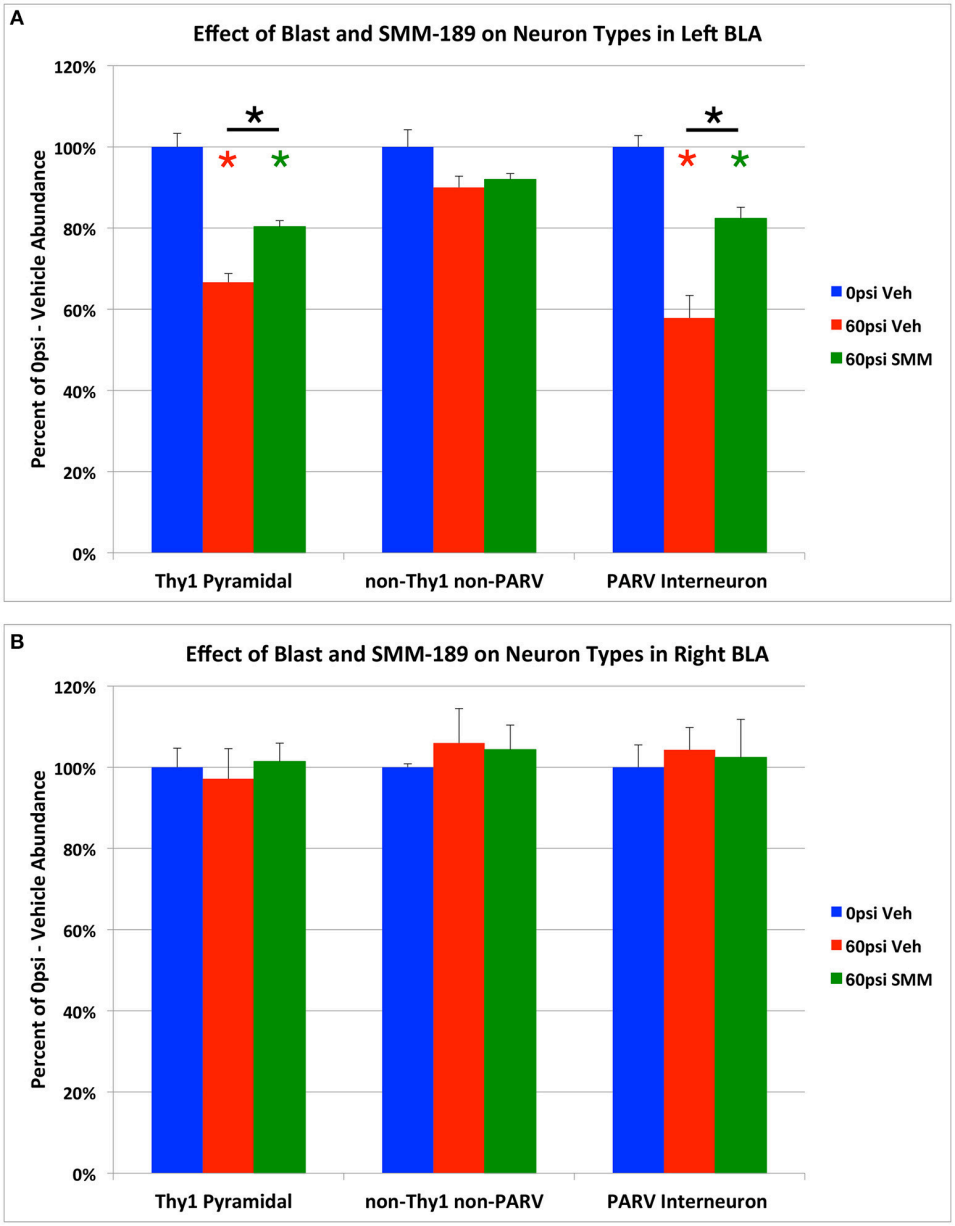
**Graphs showing the effect of TBI and SMM-189 on neuron types in left BLA (A), and in right BLA (B)**. Thy1+ pyramidal neurons and PARV+ interneurons are decreased in number in the left BLA of 60-psi blast mice, and are both rescued in the SMM-189 treated mice. By contrast, there was no significant change in the non-Thy1/non-PARV neurons of left BLA (which are mainly fear-promoting pyramidal neurons) of BLA after TBI. Red asterisks indicate a significant difference between vehicle-treated 60-psi mice and vehicle-treated 0-psi mice. Green asterisks indicate a significant difference between SMM-189 and vehicle-treated 0-psi mice. Asterisks above bars spanning the SMM-189 and vehicle-treated 60-psi mice columns indicate a significant difference between these two conditions. No significant changes in Thy1+ pyramidal neurons, PARV+ inhibitory interneurons, or non-Thy1/non-PARV neurons were seen for the right BLA following TBI, with or without treatment **(B)**.

Blasts of 60-psi to the left side of the head resulted in 20.1% overall loss (p = 0.000069) of NeuN-immunostained neurons from the left BLA (Figures 6A,B, 7A), with no significant change in the volume of left BLA. Thy1+ pyramidal neurons showed a 33.3% loss (*p* = 0.000014) in vehicle-treated 60-psi mice compared to vehicle-treated sham blast mice (Figures 6D,E, 7A). Similarly, PARV+ interneurons showed a 42.1% neuron loss (*p* = 0.00001; Figures 6G,H, 7A). As the Thy1+ pyramidal neurons and the PARV+ interneurons of BLA are involved in reduction and extinction of learned fear (Myers et al., 2006; Herry et al., 2008; Heldt et al., 2012; Jasnow et al., 2013), their loss is likely to contribute to the increased learned contextual fear and diminished fear extinction exhibited by mice that we had subjected to 50–60 psi blasts (Heldt et al., 2014; Reiner et al., 2015). By contrast, the remaining neurons (i.e., Thy1-negative/PARV-negative), which largely constitute the Thy1-negative pyramidal neurons of BLA, showed only a small reduction that was not statistically significant (~10% loss; *p* = 0.109; Figure 7A). Since the non-Thy1+ pyramidal neurons promote fear, their preferential preservation, together with the substantial loss of the Thy1-enriched fear-reducing pyramidal neurons may explain why TBI increases fear in our model (Haubensak et al., 2010; Herry and Johansen, 2014; Lüthi and Lüscher, 2014).

We found that SMM-189 treatment significantly rescued the TBI-related loss of both Thy1+ neurons and PARV+ interneurons, returning these to 80.3 and to 82.4% of sham abundance, respectively (Figures 6D–I, 7A). The abundance of Thy1+ BLA neurons in SMM-189 treated 60-psi mice was significantly greater than in vehicle-treated 60-psi mice (*p* = 0.010), although it remained less than in vehicle-treated 0-psi mice (*p* = 0.001). Similarly, the abundance of PARV+ BLA neurons in SMM-189 treated 60-psi mice was greater than in vehicle-treated 60-psi mice (*p* = 0.005), but still less than in vehicle-treated 0-psi mice (*p* = 0.034). SMM-189 trended toward rescue of overall BLA neuron loss, as reflected in the NeuN immunolabeling (Figures 6A–C), reducing it from 79.9% to 87.2% (*p* = 0.054842). By contrast, with SMM-189 treatment, the Thy1-negative/PARV-negative BLA neurons were found to be 92.0% of sham-vehicle abundance—not significantly more than in vehicle-treated 60-psi mice (*p* = 1.000), nor significantly less than in vehicle-treated 0-psi mice (*p* = 0.240; Figure 7A).

In contrast to left BLA, no significant loss was observed in the right BLA for the Thy1+ neurons (*p* = 1.000), the PARV+ neurons (*p* = 1.000), or the Thy1-negative/PARV-negative NeuN neurons (*p* = 0.611; Figure 7B), or for right BLA volume as measured in the sections used for Thy1, PARV or NeuN neuron counts. Finally, the abundance of each of these three neuron types in right BLA did not differ significantly between SMM-189 treated blast mice and the sham mice.

## Discussion

### Neuronal loss with TBI

#### Cerebral cortex and striatum

We have previously reported that our model of mild TBI produces diffuse axonal injury, as evidenced by the presence of swollen axonal bulbs at a few days, and degenerating axons at 1–2 weeks after blast (Heldt et al., 2014; Guley et al., 2016). As in humans and in other animal models of TBI (Petras et al., 1997; Shitaka et al., 2001; Koliatsos et al., 2011; MacDonald et al., 2011; Johnson et al., 2013; Morey et al., 2013; Xu et al., 2016), damaged axons were most prevalent in the major white fiber tracts, where their parallel arrangement is thought to make them especially vulnerable to breakage by the stretch and shear forces produced when the blast pressure wave is conducted through the brain parenchyma (Smith et al., 2013b). In turn, this widespread axonal damage may contribute to the apparently diffuse loss of neurons we report here for the cerebral cortex and striatum, as well as to the neuron loss and atrophy observed in cortex and striatum after closed-head TBI in humans (Wilde et al., 2007; Maxwell et al., 2010; Leunissen et al., 2014) and after mild TBI in animal models (Smith et al., 1997; Cho et al., 2013; Sajja et al., 2013; Goddeyne et al., 2015). The observed neuronal loss in our study was not obviously localized to a restricted region and was not evident without stereological neuron counts. It thus seems unlikely that neuronal loss occurred disproportionately in a particular region (e.g., frontal, visual, somatosensory, motor, or medial vs. lateral) or layer of cerebral cortex, or in a particular region or compartment of the striatum. However, additional analysis and the use of cell-type specific markers would be required to determine if there was any loss of interneurons and/or any preferential loss of a specific subpopulation(s) of projection neurons from cortex or striatum (Reiner et al., 1998; Hattox and Nelson, 2007; DeFelipe et al., 2013; Deng et al., 2015). Prior studies have reported that projection neurons in cortex and striatum are vulnerable to TBI (Maxwell et al., 2010; Bales et al., 2011), but interneurons, at least those in the hippocampus, have been reported to also be vulnerable to TBI (Lowenstein et al., 1992; Hicks et al., 1993; Smith et al., 1997; Tsuda et al., 2016).

The loss of neurons in the cerebral cortex and striatum was not only seemingly distributed throughout each, but also was similar in extent for the two sides of the brain. It may be that the bilateral neuronal loss stems from bilateral axonal injury, as both cortex and striatum are composed primarily of projection neurons possessing long axons, which travel in the corpus callosum or pyramidal tract and in the ansa lenticularis or striatonigral tract, respectively (Reiner et al., 1998; Hattox and Nelson, 2007; Deng et al., 2015). The fact that the pyramidal tract, ansa lenticularis and striatonigral tract are juxtaposed at their entry into the thalamus and midbrain may make their axons subject to similar tensile and shear forces at this level of the brain, and thus bilaterally vulnerable. A possible reason for the greater extent of neuron loss we find for the striatum as compared to cortex (~30 vs. ~20%) may relate to the higher proportion of projection neurons in the striatum (~95%) as compared to the cerebral cortex (~80%) (Peters et al., 1985; Reiner et al., 1998; Bartolini et al., 2013), with all striatal projection neurons, but only about half of all cortical projection neurons, having axons that follow a longitudinal trajectory in the brain. Longitudinally running axons appear to be more susceptible to damage in our TBI model, in that we observed axonal bulbs and silver-stained degenerating axons more frequently in the pyramidal and optic tracts than in the corpus callosum (Heldt et al., 2014; Guley et al., 2016). Similarly, the pyramidal and optic tracts are especially vulnerable to axonal injury with closed skull impact TBI, for example the Marmarou impact acceleration approach, which also subjects axons to stretch and shear forces (Kallakuri et al., 2012; Zakaria et al., 2012). Based on published computer models of TBI biomechanics (Taylor and Ford, 2009; Laksari et al., 2014), the blast wave created using our system would be expected to also compress the brain, first on the targeted left side and then on the contrecoup side as the brain moves within the skull, and this may further contribute to the generalized bilateral neuron loss we found. Relatively little is known, however, about the long-term deleterious consequences of compressive forces on axons and neuronal cell bodies (Meaney and Smith, 2011).

It should be noted that we previously reported an ~10% loss of axons in the dorsal corticospinal (CST) axons at thoracic spinal cord levels on the right side as compared to the left side following left side cranial blast (Guley et al., 2016). We also observed an asymmetry in signs of axonal injury along the descending pyramidal tracts at the level of the pons, with more on the left side (Guley et al., 2016). As axons arising from corticospinal motoneurons (CSMNs) on the left side of the brain cross the midline at the spinomedullary junction, the results for CST axons might suggest that damage was limited to the left side of motor cortex, and at first glance, appear to contradict the bilateral loss of cortical neurons reported here. However, our analysis of axon loss, which compared the areas of axons immunostained for protein kinase C gamma on the two sides, would not rule out the possibility that CST axons on the left side of the spinal cord were also lost, just that loss was greater for the right side. Moreover, CSMNs are estimated to comprise less than 5% of the neurons in motor cortex (Özdinler et al., 2011) and so a preferential loss of CSMNs from left but not right motor cortex, would not significantly affect the overall cortical counts reported here.

The injury produced in our blast model is classified as mild TBI, based on the absence of obvious contusion or hemorrhaging, the rapidity with which animals awaken from anesthesia after TBI, and the absence of any post-TBI torpor. It thus is surprising to find that 20–30% of neurons had been lost by 2–3 months after the injury. Few other studies of mild TBI have, however, examined this issue. Although some published reports have shown dying neurons by immunolabeling for activated caspase, or staining using fluorojade or TUNEL (e.g., Raghupathi et al., 2002; Longhi et al., 2005; Sajja et al., 2015), these approaches do not provide information about the ultimate extent of neuronal loss. Further, in many cases, histological examination of the brain has been limited to 10 days or less after the TBI event. The main exceptions are several studies in which researchers, having observed extensive axonal injury in the optic tract and nerve, have then counted surviving retinal ganglion cells (Tzekov et al., 2014; Xu et al., 2016). For example, Xu et al. (2016) reported 30% retinal ganglion cell loss by 10 weeks after impact TBI. Interestingly this is similar to the amount of loss we found for the striatum, with its 95% complement of projection neurons, and is consistent with observations that the majority of axons are not injured by mild TBI. Other studies have shown substantial cortical thinning, striatal atrophy, and up to 40% hippocampal neuron loss as long-term consequences of contusive cortical injury (Baldwin et al., 1997; Smith et al., 1997). This type of TBI is, however, severe in that the skull is open and the exposed dura is impacted. Thus, to the best of our knowledge, no studies other than our own have examined the consequence of mild TBI for neuron loss in the brain. It may be that the 20–30% loss we observed occurs over a time frame of several weeks and that diffuse neuron loss goes undetected unless stereological analysis is performed.

#### Basolateral amygdala

Neuronal loss in BLA varied in several ways from that in cerebral cortex and striatum. Most strikingly, it was limited to the targeted side of the brain rather than occurring bilaterally. BLA is situated slightly deep and anterior to the center of the area targeted by the blast region, which corresponds to the thalamus. The presence of the fluid-filled lateral and third ventricles at this level may alter the transmission of tensile, shear, and compressive forces from one side of the brain to the other, and may thereby “protect” deeper neural structures such as the amygdala on the non-blast side, as may also the transverse course of its efferent axons (Gupta and Przekwas, 2013). In addition, the left BLA neuron loss involved 33.3% reduction in one population of pyramidal projection neurons (Thy1+) but no significant loss in the other major population of BLA pyramidal projection neurons (i.e., the Thy1-negative), as well as a 42.1% loss of PARV interneurons. The present results are consistent with our prior report of Thy1 neuron loss in BLA after mild TBI (Heldt et al., 2014; Reiner et al., 2015), and with reports of GABAergic neuron loss from BLA after controlled cortical impact TBI (Almeida-Suhett et al., 2014). Although we did not observe neuron loss in the right BLA, it is nonetheless possible that functional changes occurred in Thy1 and PARV neurons in right BLA, given that neurons can be dysfunctional after brain injury or in neurodegenerative diseases, in the absence of or prior to overt neuron loss (Cohen et al., 2007; Smith et al., 2013a; Deng et al., 2014).

#### Mechanisms of neuron loss

Taken together, our results for cortex and striatum suggest that axonal injury may contribute to subsequent neuron death after TBI, whereas our results for PARV+ interneurons of BLA indicate that axonal injury is not necessary for neuronal loss for at least some neuron types in our model. Moreover, the basis of the differential vulnerability of Thy1+ vs. Thy1-negative pyramidal neurons of left BLA is uncertain, as both send axons to the nearby central amygdala, as well as more distantly to the medial prefrontal cortex (Lüthi and Lüscher, 2014). A variety of cellular characteristics confer susceptibility to neuronal death after trauma, for example, a limited ability to buffer calcium or a high vulnerability to excitotoxic or oxidative injury (McGinn and Povlishock, 2015; Jayakumar et al., 2016; Tovar-y-Romo et al., 2016). In addition, neuroinflammation often has detrimental effects in the aftermath of TBI (Brown and Vilalta, 2015; Loane and Kumar, 2016) and is likely to contribute to the neuron loss we find here. Consistent with this possibility, neuron loss appears to be progressive in our model, as we previously found ~12% cortical neuron loss at 45 days after blast (Guley et al., 2016), as compared to the ~20% loss at 2–3 months post-blast reported here. We do not know if cortical and/or striatal neurons continue to die beyond the 3-month time point and, if so, at what rate. Moreover, we do we know if neuronal death occurs and is progressive in other regions as well. The additional loss of ~8% of cortical neurons between 6 and 10 weeks after the initial traumatic event cannot readily be explained by damage to the neurons themselves. Moreover, as will be discussed in more detail below, that SMM-189 treatment rescued roughly half of the neurons that would have otherwise been lost, suggests that the rescued neurons died as consequence of neuroinflammatory processes. It is possible that yet more neurons may be capable of rescue, since we do not know if the dose or timing of SMM-189 treatment we used could be further optimized.

### Neuronal loss and rescue in cerebral cortex and striatum and relation to motor deficits

The neuronal loss we find in cerebral cortex and striatum at 3–4 months is correlated with the rotarod deficits these mice exhibited at 2 weeks post blast (Guley et al., 2016), indicating that the injury severity in the 2 weeks after blast and its rescue with SMM-189 predict the deficits and ultimate neuron loss. Consistent with this, treatment for 2 weeks after blast with the CB2 inverse agonist SMM-189 rescues about half the total number of cortical and striatal neurons that would have otherwise died, and significantly reduces rotarod deficits (Reiner et al., 2015). Whether the rescue of cortical neurons with SMM-189 treatment is the cause or consequence of the rescue of CST axons that we previously reported (Reiner et al., 2015) is uncertain. The present results extend on our prior findings, in that they show that rescue of sensorimotor deficits causing impaired rotarod performance may be due to the preservation of cortical and striatal neurons. Much of the area of the cortex for which counts were obtained corresponds to somatosensory and motor regions and staying on rotarod requires sensory-motor coordination. Similarly, the correlation of striatal neuron abundance with rotarod performance is consistent with role of striatum, as it is important in movement initiation, action sequence coordination, and motor learning (Deng et al., 2015; Yttri and Dudman, 2016). It is likely that TBI in our model produces cortical neuron loss throughout the entire cortex and deficits in functions that we have not assayed, such as in somesthesis, audition, and memory. Consistent with this, we have detected electrophysiological abnormalities in prefrontal cortex, hippocampus, visual cortex and somatosensory cortex as early as 1 month after blast (Liu et al., 2016). Whether deficits in memory and in sensory modalities other than vision occur after blast in our model and are also rescued by SMM-189 treatment requires further study.

### Neuronal loss and rescue in BLA and relation to fearfulness

We previously reported that mild TBI with our model causes an increase in fearfulness as assessed in an auditory fear conditioning paradigm, as well as a reduction in the Thy1+ neurons of BLA (as determined from confocal microscopy), and that both are rescued with SMM-189 treatment (Reiner et al., 2015). The increased fearfulness manifests as both an increase in freezing (freezing being the standard indicator of fear in rodents) to a learned auditory fear stimulus that signals impending shock, and an enhancement of contextual fear when the mice are placed in the fear-training chamber (Heldt et al., 2014). Moreover, the TBI mice exhibit greater resistance to extinction of the learned fear, in that more extinction trials are needed to extinguish the learned fear response than is the case for sham blast mice. The fear is perseverative and progressive, as contextual fear persists and learned fear is even more resistant to extinction in TBI mice at 1 year after blast (Heck et al., 2015). In the present study, stereological neuron counts showed a 33.3% loss of the Thy1+ fear-suppressing pyramidal neurons and a 42.1% loss of PARV+ fear-suppressing interneurons in BLA, but no significant loss of the fear-promoting Thy1-negative pyramidal neurons of BLA. Together, these findings help explain the increased fearfulness occurring in our TBI model, as preferential loss of fear-suppressing neurons should lead to increased fear responses. Moreover, SMM-189 treatment significantly reduced the Thy1 and PARV neuron loss in BLA, with the rescue of these neuron types most likely representing the means by which SMM-189 reduces post-TBI fearfulness.

**Figure 8:**
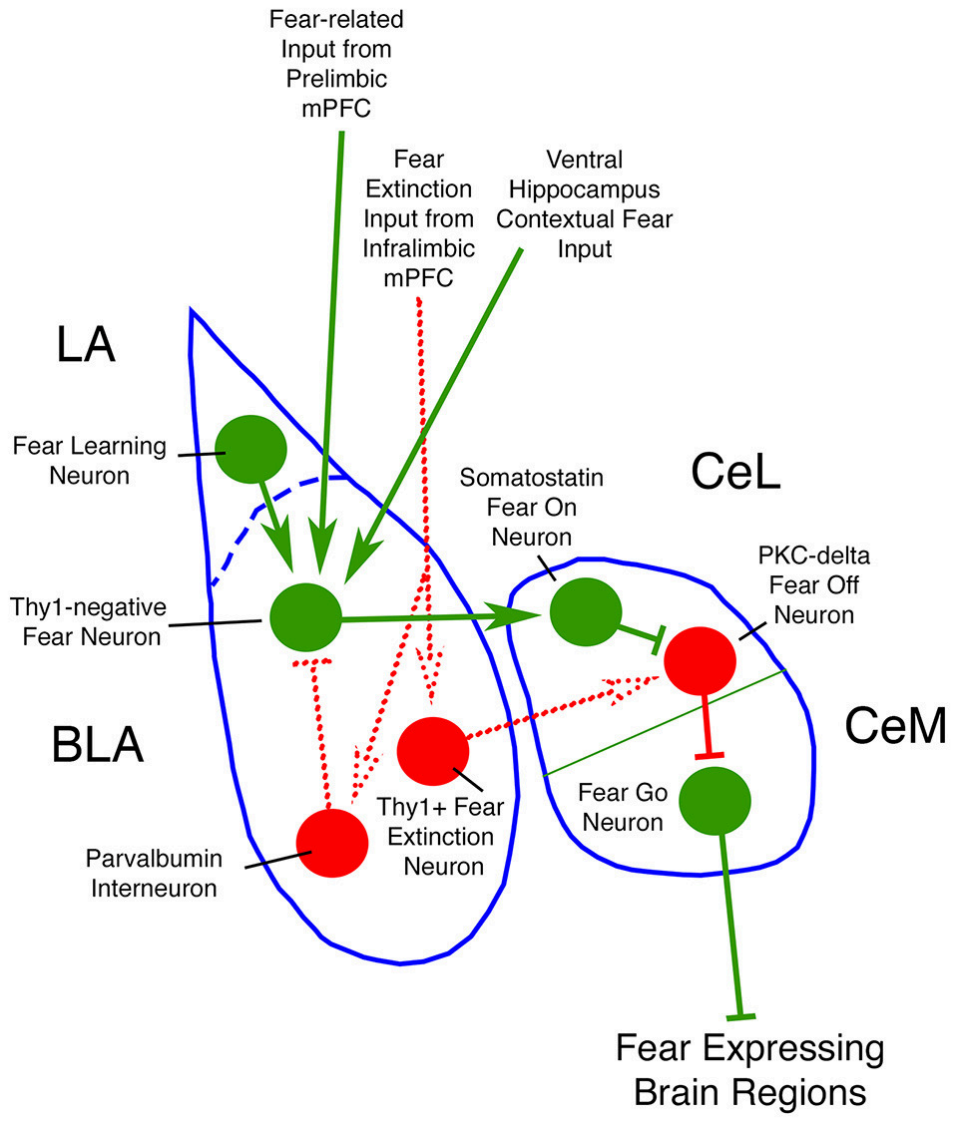
**Circuit diagram of neuron types of amygdala of interest to the present study**. Neurons and inputs shown in green promote fear, while those shown in red reduce fear. Dotted lines represent connections that are less firmly established in the literature than are the connections shown by solid lines.

The amygdala contributes to fear via the interplay of three cell groups, BLA, the lateral anterior nucleus (LA), and the central nucleus (CeA) (Figure 8; Ehrlich et al., 2009; Ciocchi et al., 2010; Morozov et al., 2011; Janak and Tye, 2015). The LA and BLA each contain about 90% excitatory pyramidal neurons and about 10% GABAergic inhibitory interneurons. The CeA consists mainly of GABAergic inhibitory neurons, some of which are projection neurons while others are local circuit neurons. Fear associations are learned by LA neurons via their inputs from sensory and pain regions. Learned fear is then signaled to BLA neurons that activate an intrinsic fear circuit in the CeA that provides output to various brainstem sites mediating the affective, autonomic and motor components of fear behavior. This fear output from BLA appears to occur via Thy1-negative pyramidal neurons that receive input from LA neurons that learn to associate a neutral stimulus with a given aversive event. These Thy1-negative fear-promoting neurons in BLA project to GABAergic neurons of CeA that promote fear (Herry et al., 2008; Haubensak et al., 2010; Jasnow et al., 2013; Herry and Johansen, 2014; Lüthi and Lüscher, 2014). By contrast, the Thy1-enriched pyramidal neurons in BLA suppress fear and project to CeA neurons that suppress fear. BLA in rodents appears to receive its main excitatory drive via cortical inputs from different parts of the ventral medial prefrontal cortex (mPFC), which is specialized for the integration of emotional states with environmental stimuli. Projections from the fear-promoting prelimbic part of mPFC mainly target the fear-promoting pyramidal BLA neurons, while the fear-reducing infralimbic part of mPFC targets fear-suppressing pyramidal neurons and GABAergic inhibitory BLA interneurons (Rosenkranz and Grace, 2001; Paré et al., 2004; Herry et al., 2008; Jasnow et al., 2013). To better understand how mild TBI increases fearfulness, and how SMM-189 rescues this deficit, it would be valuable to know how the inputs from the prelimbic and infralimbic parts of mPFC, as well as the input from ventral hippocampus, to BLA are affected by mild TBI.

The BLA contains four major types of GABAergic inhibitory interneurons constituting about 10% of its total neuronal population - a parvalbuminergic (PARV) type, a somatostatinergic (SST) type, a large-sized type containing cholecystokinin (CCK), and a small-sized type containing vasoactive intestinal polypeptide (VIP) (Rainnie et al., 2006; Muller et al., 2007). The PARV interneurons primarily target pyramidal BLA neurons, making up half of their inhibitory input (Muller et al., 2006; Wolff et al., 2014), with the other types of interneurons providing the other half (Mascagni and McDonald, 2003; Muller et al., 2003, 2007). Both the PARV interneurons (Godavarthi et al., 2014; Wolff et al., 2014) and the CCK interneurons (Truitt et al., 2009) appear to reduce fear and anxiety, while the SST interneurons appear to increase fear (Wolff et al., 2014). One simple way in which PARV and CCK interneurons may reduce fear and anxiety, and SST neurons may increase fear, is if the PARV and CCK interneurons preferentially innervate the Thy1-negative fear-promoting BLA neurons, and SST interneurons preferentially innervate the Thy1-rich fear suppressing neurons of BLA. Future studies will be needed to characterize the connectivity of these other interneuron types with pyramidal neurons, and to determine how each type is affected by mild TBI, to more fully understand how mild TBI increases fearfulness, and how SMM-189 treatment produces its rescue of fearfulness.

### Microglial activation and SMM-189 therapy

We previously showed that the axonal injury in major fiber tracts resulting from blast in our TBI model is accompanied by microglial activation along those fiber tracts and in brain regions where the damaged axons terminate (Guley et al., 2016). It is known that injured axons and their myelin sheaths release molecules that can bind to damage-associated molecular pattern molecule receptors, which include Toll-like receptors expressed on microglia, thereby activating them (Kumar and Loane, 2012). The activated microglia then typically release pro-inflammatory factors that have toxic effects on nearby neurons, may help recruit other immune cells to the region, and can compromise the integrity of the blood-brain barrier, leaving the central nervous system vulnerable to molecules in the systemic circulation (Brown and Vilalta, 2015; Loane and Kumar, 2016). Thus, an initial injury has the potential to lead to a continuing cascade of deleterious effects. Our present finding that blast TBI yields substantial neuron loss in cortex, striatum, and amygdala thus indicates that the initial trauma and the cascade it initiates leads not only to axonal injury but also neuron loss, perhaps in some cases secondary to axonal injury.

The rapid upregulation of CB2 by microglia after TBI allows them to be specifically targeted by SMM-189 for therapeutic purposes. Our prior *in vitro* analysis has shown that SMM-189 biases microglia from a pro-inflammatory M1 phenotype to a pro-healing M2 phenotype (Presley et al., 2015; Reiner et al., 2015). In addition, we have previously shown that treatment with SMM-189 reduces microglial activation in the right optic tract and retina, as assessed with IBA1 immunolabeling, and biases microglia away from the M1 state toward the M2 state, as indicated by increased pCREB immunolabeling (Reiner et al., 2015; Guley et al., 2016). In the present study, we have extended this observation to several additional brain regions, including BLA, the striatum, the terminal projection field of the striatum in the substantia nigra pars reticulata, and the white matter tract passing from cerebral cortex to brainstem. In all cases, microglia in these regions in blast-only mice rarely possessed evident nuclear pCREB, whereas in the SMM-189-treated mice, all the microglia showed prominent nuclear pCREB staining. Interestingly, the CD68 immunolabeling revealed some rod-like microglia in the internal capsule in mice with blast plus SMM-189, all with prominent nuclear pCREB. The presence of rod-like microglia is of interest because we have previously shown that SMM-189 promotes a rod-like morphology in microglia *in vitro* (Presley et al., 2015). Moreover, others have demonstrated that rod-shaped microglia show increased M2 marker expression after stable alignment with their substrate (Tam and Ma, 2014), suggesting that such microglia play a role in repairing nervous system damage.

Overall then, our results show that SMM-189 acts on brain microglia and biases them to the beneficial M2 state in the brain regions (and/or their fiber tracts) where SMM-189 rescues TBI-related neuron loss. Our present finding that SMM-189 treatment for the 2 weeks after blast yields substantial neuron rescue in cortex, striatum, and amygdala thus indicates that M1 microglial activation during the aftermath of mild TBI worsens the outcome, and that biasing toward M2 microglial activation during this same time period allows the survival of many of the neurons that would have otherwise died. It is likely that the observed neuronal rescue contributed to the reduction of motor deficits and fearfulness that mild TBI normally produces. Note that treatment with a CB2 agonist instead would reduce M1 activation but not promote M2 activation, which may explain why CB2 agonists have not consistently shown strong benefit in treating TBI (Mechoulam et al., 2002; Meyer et al., 2010; Elliott et al., 2011; Amenta et al., 2012, 2014; Firsching et al., 2012). Our results thus support further testing of CB2 inverse agonists as a useful therapeutic approach for reducing neural injury and functional deficits after mild TBI. It is possible that further optimization of dosage and timing would produce even greater benefit than we found here and that there is a critical time window during which treatment would be most effective. It will also be important to extend testing the efficacy of SMM-189 to additional neuronal populations and functions in our TBI model, as well as determine its usefulness in other TBI models.

## Author contributions

WB, HR, YD, ND, and NG carried out research. BM developed, characterized and provided SMM-189. WB, HR, YD, MH, and AR analyzed data. BM, MH, and AR wrote the manuscript.

## Funding

Supported by the Neuroscience Institute and the Office of the Dean of the College of Medicine, both at UTHSC, The Methodist Hospitals Endowed Professorship in Neuroscience (AR), NIH grant NS-081370 (AR), and the College of Pharmacy at UTHSC (BM).

### Conflict of interest statement

The authors declare that the research was conducted in the absence of any commercial or financial relationships that could be construed as a potential conflict of interest.

## References

[B1] Almeida-Suhett C. P. Prager E. M. Pidoplichko V. Figueiredo T. H. Marini A. M. Li Z.. (2014). Reduced GABAergic inhibition in the basolateral amygdala and the development of anxiety-like behaviors after mild traumatic brain injury. PLoS ONE 9:e102627. 10.1371/journal.pone.0102627 25047645 PMC4105413

[B2] Amenta P. S. Jallo J. A. Tuma R. F. Hooper D. C. Elliott M. B. (2014). Cannabinoid receptor type-2 stimulation, blockade, and deletion alter the vascular inflammatory responses to traumatic brain injury. J. Neuroinflammation 11, 191. 10.1186/s12974-014-0191-6 25416141 PMC4248435

[B3] Amenta P. S. Jallo J. I. Tuma R. F. Elliott M. B. (2012). A cannabinoid type-2 receptor agonist attenuates blood-brain barrier damage and neurodegeneration in a murine model of traumatic brain injury. J. Neurosci. 90, 2293–2305. 10.1002/jnr.23114 22903455

[B4] Ashton J. C. Glass M. (2007). The cannabinoid CB_2_ receptor as a target for inflammation-dependent neurodegeneration. Curr. Neuropharmacol. 5, 73–80. 10.2174/157015907780866884 18615177 PMC2435344

[B5] Atwood B. K. Straiker A. Mackie K. (2012). CB_2_ therapeutic target-in-waiting. Prog. Neuropsychopharmacol. Biol. Psychiat. 38, 16–20. 10.1016/j.pnpbp.2011.12.001 PMC3345167 22197668

[B6] Avramescu S. Nita D. A. Timofeev I. (2009). Neocortical post-traumatic epileptogenesis is associated with loss of GABAergic neurons. J. Neurotrauma 26, 799–812. 10.1089/neu.2008.0739 19422294 PMC2735829

[B7] Baek J. H. Darlington C. L. Smith P. F. Ashton J. C. (2013). Antibody testing for brain immunohistochemistry: brain immunolabeling for the cannabinoid CB_2_, receptor. J. Neurosci. Methods 216, 87–95. 10.1016/j.jneumeth.2013.03.021 23583232

[B8] Baldwin S. A. Gibson T. Callihan C. T. Sullivan P. G. Palmer E. Scheff S. W. (1997). Neuronal cell loss in the CA3 subfield of the hippocampus following cortical contusion utilizing the optical disector method for cell counting. J. Neurotrauma 14, 385–398. 10.1089/neu.1997.14.385 9219853

[B9] Bales J. W. Yan H. Q. Ma X. Li Y. Samarasinghe R. Dixon C. E. (2011). The dopamine and cAMP regulated phosphoprotein, 32 kDa (DARPP-32) signaling pathway: a novel therapeutic target in traumatic brain injury. Exp. Neurol. 229, 300–307. 10.1016/j.expneurol.2011.02.013 21376040 PMC3110667

[B10] Bareyre F. M. Kerschensteiner M. Misgeld T. Sanes J. R. (2005). Transgenic labeling of the corticospinal tract for monitoring axonal responses to spinal cord injury. Nat. Med. 11, 1355–1360. 10.1038/nm1331 16286922

[B11] Bartolini G. Ciceri G. Marín O. (2013). Integration of GABAergic interneurons into cortical cell assemblies: lessons from embryos and adults. Neuron 79, 849–864. 10.1016/j.neuron.2013.08.014 24012001

[B12] Bazarian J. J. Donnelly K. Peterson D. R. Warner G. C. Zhu T. Zhong J. (2013). The relation between posttraumatic stress disorder and mild traumatic brain injury acquired during operations Enduring Freedom and Iraqi Freedom. J. Head Trauma Rehabil. 28, 1–12. 10.1097/HTR.0b013e318256d3d3 22647965

[B13] Benito C. Nú-ez E. Tolón R. M. Carrier E. J. Rábano A. Hillard C. J.. (2003). Cannabinoid CB_2_ receptors and fatty acid amide hydrolase are selectively overexpressed in neuritic plaque-associated glia in Alzheimer's disease brains. J. Neurosci. 23, 11136–11141. 14657172 10.1523/JNEUROSCI.23-35-11136.2003 PMC6741043

[B14] Brown G. C. Vilalta A. (2015). How microglia kill neurons. Brain Res. 1628(Pt B), 288–297. 10.1016/j.brainres.2015.08.031 26341532

[B15] Cao T. Thomas T. C. Ziebell J. M. Pauly J. R. Lifshitz J. (2012). Morphological and genetic activation of microglia after diffuse traumatic brain injury in the rat. Neuroscience 225, 65–75. 10.1016/j.neuroscience.2012.08.058 22960311 PMC3489473

[B16] Cherry J. D. Olschowka J. A. O'Banion M. K. (2014). Neuroinflammation and M2 microglia: the good, the bad, and the inflamed. J. Neuroinflammation 11:98. 10.1186/1742-2094-11-98 24889886 PMC4060849

[B17] Cho H. J. Sajja V. S. Vandevord P. J. Lee Y. W. (2013). Blast induces oxidative stress, inflammation, neuronal loss and subsequent short-term memory impairment in rats. Neuroscience 253, 9–20. 10.1016/j.neuroscience.2013.08.037 23999126

[B18] Ciocchi S. Herry C. Grenier F. Wolff S. B. Letzkus J. J. Vlachos I.. (2010). Encoding of conditioned fear in central amygdala inhibitory circuits. Nature 468, 277–282. 10.1038/nature09559 21068837

[B19] Cohen A. S. Pfister B. J. Schwarzbach E. Grady M. S. Goforth P. B. Satin L. S. (2007). Injury-induced alterations in CNS electrophysiology. Prog. Brain Res. 161, 143–169. 10.1016/S0079-6123(06)61010-8 17618975

[B20] Das M. Mohapatra S. Mohapatra S. S. (2012). New perspectives on central and peripheral immune responses to acute traumatic brain injury. J. Neuroinflammation 9:236. 10.1186/1742-2094-9-236 23061919 PMC3526406

[B21] DeFelipe J. López-Cruz P. L. Benavides-Piccione R. Bielza C. Larra-aga P. Anderson S.. (2013). New insights into the classification and nomenclature of cortical GABAergic interneurons. Nat. Rev. Neurosci. 14, 202–216. 10.1038/nrn3444 23385869 PMC3619199

[B22] Deng Y. Lanciego J. Goff L. K. Coulon P. Salin P. Kachidian P.. (2015). Differential organization of cortical inputs to striatal projection neurons of the matrix compartment in rats. Front. Syst. Neurosci. 9:51. 10.3389/fnsys.2015.00051 25926776 PMC4396197

[B23] Deng Y. P. Xie J. P. Wang H. B. Lei W. L. Chen Q. Reiner A. (2007). Differential localization of the GluR1 and GluR2 subunits of the AMPA-type glutamate receptor among striatal neuron types in rats. J. Chem. Neuroanat. 33, 167–192. 10.1016/j.jchemneu.2007.02.008 17446041 PMC1993922

[B24] Deng Y. Wong T. Wan J. Y. Reiner A. (2014). Differential loss of thalamostriatal and corticostriatal input to striatal projection neuron types prior to overt motor symptoms in the Q140 knock-in mouse model of Huntington's disease. Front. Syst. Neurosci. 8:198. 10.3389/fnsys.2014.00198 25360089 PMC4197654

[B25] Donat C. K. Fischer F. Walter B. Deuther-Conrad W. Brodhun M. Bauer R.. (2014). Early increase of cannabinoid receptor density after experimental traumatic brain injury in the newborn piglet. Acta Neurobiol. Exp. (Wars) 74, 197–210. 24993629 10.55782/ane-2014-1985

[B26] Ehrlich I. Humeau Y. Grenier F. Ciocchi S. Herry C. Lüthi A. (2009). Amygdala inhibitory circuits and the control of fear memory. Neuron 62, 757–771. 10.1016/j.neuron.2009.05.026 19555645

[B27] Elliott M. B. Tuma R. F. Amenta P. S. Barbe M. F. Jallo J. I. (2011). Acute effects of a selective cannabinoid-2 receptor agonist on neuroinflammation in a model of traumatic brain injury. J. Neurotrauma 28, 973–981. 10.1089/neu.2010.1672 21332427

[B28] Faul M. Xu L. Wald M. M. Coronado VG (2010). Traumatic Brain Injury in the United States: Emergency Department Visits, Hospitalizations, and Deaths. Atlanta, GA: Centers for Disease Control and Prevention. National Center for Injury Prevention and Control.

[B29] Firsching R. Piek J. Skalej M. Rohde V. Schmidt U. Striggow F.. (2012). Early survival of comatose patients after severe traumatic brain injury with the dual cannabinoid CB1/CB2 receptor agonist KN38-7271: a randomized, double-blind, placebo-controlled phase II trial. J. Neurol. Surg. A Cent. Eur. Neurosurg. 73, 204–216. 10.1055/s-0032-1304815 22696266

[B30] Fujinaga M. Kumata K. Yanamoto K. Kawamura K. Yamasaki T. Yui J.. (2010). Radiosynthesis of novel carbon-11- labeled triaryl ligands for cannabinoid-type 2 receptor. Bioorg. Med. Chem. Lett. 20, 1565–1568. 10.1016/j.bmcl.2010.01.074 20137936

[B31] Gelderblom H. Verweij J. Nooter K. Sparreboom A. (2001). Cremophor EL: the drawbacks and advantages of vehicle selection for drug formulation. Eur J Cancer 37, 1590–1598. 10.1016/S0959-8049(01)00171-X 11527683

[B32] Godavarthi S. K. Sharma A. Jana N. R. (2014). Reversal of reduced parvalbumin neurons in hippocampus and amygdala of Angelman syndrome model mice by chronic treatment of fluoxetine. J. Neurochem. 130, 444–454. 10.1111/jnc.12726 24678582

[B33] Goddeyne C. Nichols J. Wu C. Anderson T. (2015). Repetitive mild traumatic brain injury induces ventriculomegaly and cortical thinning in juvenile rats. J. Neurophysiol. 113, 3268–3280. 10.1152/jn.00970.2014 25695652 PMC4440238

[B34] Gorski J. A. Talley T. Qiu M. Puelles L. Rubenstein J. L. Jones K. R. (2002). Cortical excitatory neurons and glia, but not GABAergic neurons, are produced in the emx1-expressing lineage. J. Neurosci. 22, 6309–6314. 12151506 10.1523/JNEUROSCI.22-15-06309.2002 PMC6758181

[B35] Guley N. H. Rogers J. T. Del Mar N. A. Deng Y. Islam R. M. D'Surney L.. (2016). A novel closed-head model of mild traumatic brain injury using focal primary overpressure blast to the cranium in mice. J. Neurotrauma 33, 403–422. 10.1089/neu.2015.3886 26414413 PMC4761824

[B36] Gupta R. K. Przekwas A. (2013). Mathematical models of blast-induced TBI: current status, challenges, and prospects. Front. Neurol. 4:59. 10.3389/fneur.2013.00059 23755039 PMC3667273

[B37] Hattox A. M. Nelson S. B. (2007). Layer V neurons in mouse cortex projecting to different targets have distinct physiological properties. J. Neurophysiol. 98, 3330–3340. 10.1152/jn.00397.2007 17898147

[B38] Haubensak W. Kunwar P. S. Cai H. Ciocchi S. Wall N. R. Ponnusamy R.. (2010). Genetic dissection of an amygdala microcircuit that gates conditioned fear. Nature 468, 270–276. 10.1038/nature09553 21068836 PMC3597095

[B39] Heck D. H. Liu Y. Honig M. G. Heldt S. Del Mar N. Guley N. H. (2015). Abnormalities in coherence of local field potential oscillations in medial prefrontal cortex are linked to lasting perseverative depression and fear following mild traumatic brain injury in a mouse model. Soc. Neurosci. Abst. 589.13.

[B40] Heldt S. A. Elberger A. J. Deng Y. Guley N. H. Del Mar N. Rogers J.. (2014). A novel closed-head model of mild traumatic brain injury caused by primary overpressure blast to the cranium produces sustained emotional deficits in mice. Front. Neurol. 5:2. 10.3389/fneur.2014.00002 24478749 PMC3898331

[B41] Heldt S. A. Mou L. Ressler K. J. (2012). *In vivo* knockdown of GAD67 in the amygdala disrupts fear extinction and the anxiolytic-like effect of diazepam in mice. Transl. Psychiatry 2, e181. 10.1038/tp.2012.101 23149445 PMC3565763

[B42] Herry C. Ciocchi S. Senn V. Demmou L. Müller C. Lüthi A. (2008). Switching on and off fear by distinct neuronal circuits. Nature 454, 600–606. 10.1038/nature07166 18615015

[B43] Herry C. Johansen J. P. (2014). Encoding of fear learning and memory in distributed neuronal circuits. Nat. Neurosci. 17, 1644–1654. 10.1038/nn.3869 25413091

[B44] Hicks R. R. Smith D. H. Lowenstein D. H. Saint Marie R. McIntosh T. K. (1993). Mild experimental brain injury in the rat induces cognitive deficits associated with regional neuronal loss in the hippocampus. J. Neurotrauma 10, 405–414. 10.1089/neu.1993.10.405 8145264

[B45] Janak P. H. Tye K. M. (2015). From circuits to behaviour in the amygdala. Nature 517, 284–292. 10.1038/nature14188 25592533 PMC4565157

[B46] Jasnow A. M. Ehrlich D. E. Choi D. C. Dabrowska J. Bowers M. E. McCullough K. M.. (2013). Thy1-expressing neurons in the basolateral amygdala may mediate fear inhibition. J. Neurosci. 33, 10396–10404. 10.1523/JNEUROSCI.5539-12.2013 23785152 PMC3685835

[B47] Jayakumar A. R. Bak L. K. Rama Rao K. V. Waagepetersen H. S. Schousboe A. Norenberg M. D. (2016). Neuronal cell death induced by mechanical percussion trauma in cultured neurons is not preceded by alterations in glucose, lactate and glutamine metabolism. Neurochem. Res. 41, 307–315. 10.1007/s11064-015-1801-0 26729365 PMC4775396

[B48] Johnson V. E. Stewart W. Smith D. H. (2013). Axonal pathology in traumatic brain injury. Exp. Neurol. 246, 35–43. 10.1016/j.expneurol.2012.01.013 22285252 PMC3979341

[B49] Kallakuri S. Li Y. Zhou R. Bandaru S. Zakaria N. Zhang L.. (2012). Impaired axoplasmic transport is the dominant injury induced by an impact acceleration injury device: an analysis of traumatic axonal injury in pyramidal tract and corpus callosum of rats. Brain Res. 1452, 29–38. 10.1016/j.brainres.2012.02.065 22472596

[B50] Kelley B. J. Lifshitz J. Povlishock J. T. (2007). Neuroinflammatory responses after experimental diffuse traumatic brain injury. J. Neuropathol. Exp. Neurol. 66, 989–1001. 10.1097/NEN.0b013e3181588245 17984681

[B51] Koliatsos V. E. Cernak I. Xu L. Song Y. Savonenko A. Crain B. J.. (2011). A mouse model of blast injury to brain: initial pathological, neuropathological, and behavioral characterization. J. Neuropathol. Exp. Neurol. 70, 399–416. 10.1097/NEN.0b013e3182189f06 21487304

[B52] Kordower J. H. Chu Y. Stebbins G. T. DeKosky S. T. Cochran E. J. Bennett D.. (2001). Loss and atrophy of layer II entorhinal cortex neurons in elderly people with mild cognitive impairment. Ann. Neurol. 49, 202–213 10.1002/1531-8249(20010201)49:2<202::AID-ANA40>3.0.CO;2-3 11220740

[B53] Kumar A. Loane D. J. (2012). Neuroinflammation after traumatic brain injury: opportunities for therapeutic intervention. Brain Behav. Immun. 26, 1191–1201. 10.1016/j.bbi.2012.06.008 22728326

[B54] Laksari K. Sadeghipour K. Darvish K. (2014). Mechanical response of brain tissue under blast loading. J. Mech. Behav. Biomed. Mater. 32, 132–144. 10.1016/j.jmbbm.2013.12.021 24457112

[B55] Lawrence T. Natoli G. (2011). Transcriptional regulation of macrophage polarization: enabling diversity with identity. Nat. Rev. Immunol. 11, 750–761. 10.1038/nri3088 22025054

[B56] Leunissen I. Coxon J. P. Caeyenberghs K. Michiels K. Sunaert S. Swinnen S. P. (2014). Subcortical volume analysis in traumatic brain injury: the importance of the fronto-striato-thalamic circuit in task switching. Cortex 51, 67–81. 10.1016/j.cortex.2013.10.009 24290948

[B57] Liu Y. McAfee S. S. Guley N. H. Honig M. G. Del Mar N. Bu W. (2016). Mild traumatic brain injury in mice causes region specific deficits in oscillatory neuronal activity and functional connectivity that are rescued by the novel cannabinoid type-2 receptor inverse agonist SMM-189. Soc. Neurosci. Abst. 607.14.

[B58] Loane D. J. Kumar A. (2016). Microglia in the TBI brain: the good, the bad, and the dysregulated. Exp. Neurol. 275, 316–327. 10.1016/j.expneurol.2015.08.018 26342753 PMC4689601

[B59] Longhi L. Saatman K. E. Fujimoto S. Raghupathi R. Meaney D. F. Davis J.. (2005). Temporal window of vulnerability to repetitive experimental concussive brain injury. Neurosurgery 56, 364–374. 10.1227/01.NEU.0000149008.73513.44 15670384

[B60] Lowenstein D. H. Thomas M. J. Smith D. H. McIntosh T. K. (1992). Selective vulnerability of dentate hilar neurons following traumatic brain injury: a potential mechanistic link between head trauma and disorders of the hippocampus. J. Neurosci. 12, 4846–4853. 1464770 10.1523/JNEUROSCI.12-12-04846.1992 PMC6575779

[B61] Lunn C. A. Fine J. S. Rojas-Triana A. Jackson J. V. Fan X. Kung T. T.. (2006). A novel cannabinoid peripheral cannabinoid receptor-selective inverse agonist blocks leukocyte recruitment *in vivo*. J. Pharmacol. Exp. Ther. 316, 780–788. 10.1124/jpet.105.093500 16258021

[B62] Lunn C. A. Reich E. P. Fine J. S. Lavey B. Kozlowski J. A. Hipkin R. W.. (2008). Biology and therapeutic potential of cannabinoid CB_2_ receptor inverse agonists. Br. J. Pharmacol. 153, 226–239. 10.1038/sj.bjp.0707480 17906679 PMC2219522

[B63] Lüthi A. Lüscher C. (2014). Pathological circuit function underlying addiction and anxiety disorders. Nat. Neurosci. 17, 1635–1643. 10.1038/nn.3849 25402855

[B64] MacDonald C. L. Johnson A. M. Cooper D. Nelson E. C. Werner N. J. Shimony J. S.. (2011). Detection of blast-related traumatic brain injury in U.S. military personnel. N. Engl. J. Med. 364, 2091–2100 10.1056/NEJMoa1008069 21631321 PMC3146351

[B65] Mascagni F. McDonald A. J. (2003). Immunohistochemical characterization of cholecystokinin containing neurons in the rat basolateral amygdala. Brain Res. 976, 171–184. 10.1016/S0006-8993(03)02625-8 12763251

[B66] Maxwell W. L. MacKinnon M. A. Stewart J. E. Graham D. I. (2010). Stereology of cerebral cortex after traumatic brain injury matched to the Glasgow outcome score. Brain 133(Pt 1), 139–160. 10.1093/brain/awp264 19897544

[B67] McGinn M. J. Povlishock J. T. (2015). Cellular and molecular mechanisms of injury and spontaneous recovery. Handbook Clin Neurol. 127, 67–87. 10.1016/B978-0-444-52892-6.00005-2 25702210

[B68] Meaney D. F. Smith D. H. (2011). Biomechanics of concussion. Clin. Sports Med. 30, 19–31. 10.1016/j.csm.2010.08.009 21074079 PMC3979340

[B69] Mechoulam R. Spatz M. Shohami E. (2002). Endocannabinoids and neuroprotection. Science STKE 2002:re5. 10.1126/stke.2002.129.re5 11972360

[B70] Meyer M. J. Megyesi J. Meythaler J. Murie-Fernandez M. Aubut J. A. Foley N.. (2010). Acute management of acquired brain injury part II: an evidence-based review of pharmacological interventions. Brain Injury 24, 706–721. 10.3109/02699051003692126 20376996

[B71] Morey R. A. Haswell C. C. Selgrade E. S. Massoglia D. Liu C. Weiner J.. (2013). Effects of chronic mild traumatic brain injury on white matter integrity in Iraq and Afghanistan war veterans. Hum. Brain Mapp. 34, 2986–2999. 10.1002/hbm.22117 22706988 PMC3740035

[B72] Morozov A. Sukato D. Ito W. (2011). Selective suppression of plasticity in amygdala inputs from temporal association cortex by the external capsule. J. Neurosci. 31, 339–345. 10.1523/JNEUROSCI.5537-10.2011 21209220 PMC3080111

[B73] Muller J. F. Mascagni F. McDonald A. J. (2003). Synaptic connections of distinct interneuronal subpopulations in the rat basolateral amygdalar nucleus. J. Comp. Neurol. 456, 217–236. 10.1002/cne.10435 12528187

[B74] Muller J. F. Mascagni F. McDonald A. J. (2006). Pyramidal cells of the rat basolateral amygdala: synaptology and innervation by parvalbumin-immunoreactive interneurons. J. Comp. Neurol. 494, 635–650. 10.1002/cne.20832 16374802 PMC2562221

[B75] Muller J. F. Mascagni F. McDonald A. J. (2007). Postsynaptic targets of somatostatin-containing interneurons in the rat basolateral amygdala. J. Comp. Neurol. 500, 513–529. 10.1002/cne.21185 17120289

[B76] Myers K. M. Ressler K. J. Davis M. (2006). Different mechanisms of fear extinction dependent on length of time since fear acquisition. Learn. Mem. 13, 216–223. 10.1101/lm.119806 16585797 PMC1409828

[B77] Namjoshi D. R. Good C. Cheng W. H. Panenka W. Richards D. Cripton P. A.. (2013). Towards clinical management of traumatic brain injury: a review of models and mechanisms from a biomechanical perspective. Dis. Models Mech. 6, 1325–1338. 10.1242/dmm.011320 24046354 PMC3820257

[B78] Neervannan S. (2006). Preclinical formulations for discovery and toxicology: physicochemical challenges. Expert Opin. Drug Metab. Toxicol. 2, 715–731. 10.1517/17425255.2.5.715 17014391

[B79] Özdinler P. H. Benn S. Yamamoto T. H. Güzel M. Brown RH Jr Macklis J. D. (2011). Corticospinal motor neurons and related subcerebral projection neurons undergo early and specific neurodegeneration in hSOD1G93A transgenic ALS mice. J. Neurosci. 31, 4166–4177. 10.1523/JNEUROSCI.4184-10.2011 21411657 PMC3643523

[B80] Paré D. Quirk G. J. Ledoux J. E. (2004). New vistas on amygdala networks in conditioned fear. J. Neurophysiol. 92, 1–9. 10.1152/jn.00153.2004 15212433

[B81] Patterson Z. R. Holahan M. R. (2012). Understanding the neuroinflammatory response following concussion to develop treatment strategies. Front. Cell. Neurosci. 6:58. 10.3389/fncel.2012.00058 23248582 PMC3520152

[B82] Perez-Polo J. R. Rea H. C. Johnson K. M. Parsley M. A. Unabia G. C. Xu G.. (2013). Inflammatory consequences in a rodent model of mild traumatic brain injury. J. Neurotrauma 30, 727–740. 10.1089/neu.2012.2650 23360201 PMC3941841

[B83] Peters A. Kara D. A. Harriman K. M. (1985). The neuronal composition of area 17 of rat visual cortex. III. Numerical considerations. J. Comp. Neurol. 238, 263–274. 10.1002/cne.902380303 4044915

[B84] Petras J. M. Bauman R. A. Elsayed N. M. (1997). Visual system degeneration induced by blast overpressure. Toxicology 121, 41–49. 10.1016/S0300-483X(97)03654-8 9217314

[B85] Presley C. Abidi A. Suryawanshi S. Mustafa S. Meibohm B. Moore B. M. (2015). Pre-clinical evaluation of SMM-189, a cannabinoid receptor-2 specific inverse agonist. Pharmacol. Res. Perspect. 3:e00159. 10.1002/prp2.159 26196013 PMC4506688

[B86] Raghupathi R. Conti A. C. Graham D. I. Krajewski S. Reed J. C. Grady M. S.. (2002). Mild traumatic brain injury induces apoptotic cell death in the cortex that is preceded by decreases in cellular Bcl-2 immunoreactivity. Neuroscience 110, 605–616. 10.1016/S0306-4522(01)00461-4 11934469

[B87] Rainnie D. G. Mania I. Mascagni F. McDonald A. J. (2006). Physiological and morphological characterization of parvalbumin-containing interneurons of the rat basolateral amygdala. J. Comp. Neurol. 498, 142–161. 10.1002/cne.21049 16856165

[B88] Redell J. B. Dash P. K. (2007). Traumatic brain injury stimulates hippocampal catechol-o-methyl transferase expression in microglia. Neurosci. Lett. 413, 36–41. 10.1016/j.neulet.2006.11.060 17240060 PMC1857315

[B89] Reiner A. Heldt S. A. Presley C. S. Guley N. H. Elberger A. J. Deng Y.. (2015). Emotional, sensory and motor deficits in mice after closed-head mild traumatic brain injury are alleviated by the novel CB_2_ inverse agonist SMM-189. Int. J. Mol. Sci. 16, 758–787. 10.3390/ijms16010758 25561230 PMC4307274

[B90] Reiner A. Lafferty D. C. Wang H. B. Del Mar N. Deng Y. P. (2012). The group-2 metabotropic glutamate receptor agonist LY379268 rescues neuronal, neurochemical and motor abnormalities in R6/2 Huntington's disease mice. Neurobiol. Dis. 47, 75–91. 10.1016/j.nbd.2012.03.025 22472187 PMC3376646

[B91] Reiner A. Medina L. Veenman C. L. (1998). Structural and functional evolution of the basal ganglia in vertebrates. Brain Res. Rev. 28, 235–285. 10.1016/S0165-0173(98)00016-2 9858740

[B92] Risdall J. E. Menon D. K. (2011). Traumatic brain injury. Philos Trans. R Soc. Lond B Biol. Sci. 366, 241–250. 10.1056/NEJMc1204669#SA2 21149359 PMC3013429

[B93] Rosenkranz J. A. Grace A. A. (2001). Dopamine attenuates prefrontal cortical suppression of sensory inputs to the basolateral amygdala of rats. J. Neurosci. 21, 4090–4103. 11356897 10.1523/JNEUROSCI.21-11-04090.2001 PMC6762693

[B94] Sajja V. S. Galloway M. Ghoddoussi F. Kepsel A. VandeVord P. (2013). Effects of blast-induced neurotrauma on the nucleus accumbens. J. Neurosci. Res. 91, 593–601. 10.1002/jnr.23179 23335267

[B95] Sajja V. S. Hubbard W. B. Hall C. S. Ghoddoussi F. Galloway M. P. VandeVord P. J. (2015). Enduring deficits in memory and neuronal pathology after blast induced traumatic brain injury. Sci. Rep. 5:15075. 10.1038/srep15075 26537106 PMC4633584

[B96] Schomberg D. Olson J. (2012). Immune responses of microglia in the spinal cord: contribution to pain states. Exp. Neurol. 234, 262–270. 10.1016/j.expneurol.2011.12.021 22226600

[B97] Shi L. Argenta A. E. Winseck A. K. Brunso-Bechtold J. K. (2004). Stereological quantification of GAD-67-immunoreactive neurons and boutons in the hippocampus of middle-aged and old Fischer 344 x Brown Norway rats. J. Comp. Neurol. 478, 282–291. 10.1002/cne.20303 15368530

[B98] Shitaka Y. Tran H. T. Bennett R. E. Sanchez L. Levy M. A. Dikranian K.. (2001). Repetitive closed-skull traumatic brain injury in mice causes persistent multifocal axonal injury and microglial reactivity. J. Neuropathol. Exp. Neurol. 70, 551–567. 10.1097/NEN.0b013e31821f891f 21666502 PMC3118973

[B99] Smith C. Gentleman S. M. Leclercq P. D. Murray L. S. Griffin W. S. Graham D. I.. (2013a). The neuroinflammatory response in humans after traumatic brain injury. Neuropathol. Appl. Neurobiol. 39, 654–666. 10.1111/nan.12008 23231074 PMC3833642

[B100] Smith D. H. Chen X. H. Pierce J. E. Wolf J. A. Trojanowski J. Q. Graham D. I.. (1997). Progressive atrophy and neuron death for one year following brain trauma in the rat. J. Neurotrauma 14, 715–727. 10.1089/neu.1997.14.715 9383090

[B101] Smith D. H. Hicks R. Povlishock J. T. (2013b). Therapy development for diffuse axonal injury. J. Neurotrauma 30, 307–323. 10.1089/neu.2012.2825 23252624 PMC3627407

[B102] Stella N. (2010). Cannabinoid and cannabinoid-like receptors in microglia, astrocytes and astrocytomas. Glia 58, 1017–1030. 10.1002/glia.20983 20468046 PMC2919281

[B103] Tam W. Y. Ma C. H. (2014). Bipolar/rod-shaped microglia are proliferating microglia with distinct M1/M2 phenotypes. Sci. Rep. 4:7279. 10.1038/srep07279 25452009 PMC4250916

[B104] Taylor P. A. Ford C. C. (2009). Simulation of blast-induced early-time intracranial wave physics leading to traumatic brain injury. J. Biomed. Eng. 131, 061007. 10.1115/1.3118765 19449961

[B105] Tovar-y-Romo L. B. Penagos-Puig A. Ramírez-Jarquín J. O. (2016). Endogenous recovery after brain damage: molecular mechanisms that balance neuronal life/death fate. J. Neurochem. 136, 13–27. 10.1111/jnc.13362 26376102

[B106] Truitt W. A. Johnson P. L. Dietrich A. D. Fitz S. D. Shekhar A. (2009). Anxiety-like behavior is modulated by a discrete subpopulation of interneurons in the basolateral amygdala. Neuroscience 160, 284–294. 10.1016/j.neuroscience.2009.01.083 19258024 PMC2682359

[B107] Tsuda S. Hou J. Nelson R. L. Wilkie Z. J. Mustafa G. Sinharoy A.. (2016). Prolonged hippocampal cell death following closed-head traumatic brain injury in rats. Neuroreport 27, 724–729. 10.1097/WNR.0000000000000598 27213933

[B108] Tzekov R. Quezada A. Gautier M. Biggins D. Frances C. Mouzon B.. (2014). Repetitive mild traumatic brain injury causes optic nerve and retinal damage in a mouse model. J. Neuropathol. Exp. Neurol. 73, 345–361. 10.1097/NEN.0000000000000059 24607965

[B109] Unal-Cevik I. Kilin,ç M. Gürsoy-Ozdemir Y. Gurer G. Dalkara T. (2004). Loss of NeuN immunoreactivity after cerebral ischemia does not indicate neuronal cell loss: a cautionary note. Brain Res. 1015, 169–174. 10.1016/j.brainres.2004.04.032 15223381

[B110] Wilde E. A. Bigler E. D. Hunter J. V. Fearing M. A. Scheibel R. S. Newsome M. R.. (2007). Hippocampus, amygdala, and basal ganglia morphometrics in children after moderate-to-severe traumatic brain injury. Dev. Med. Child Neurol. 49, 294–299. 10.1111/j.1469-8749.2007.00294.x 17376141

[B111] Wolff S. B. Gründemann J. Tovote P. Krabbe S. Jacobson G. A. Müller C.. (2014). Amygdala interneuron subtypes control fear learning through disinhibition. Nature 509, 453–458. 10.1038/nature13258 24814341

[B112] Xiong Y. Mahmood A. Chopp M. (2013). Animal models of traumatic brain injury. Nat. Rev. Neurosci. 14, 128–142. 10.1038/nrn3407 23329160 PMC3951995

[B113] Xu L. Nguyen J. V. Lehar M. Menon A. Rha E. Arena J.. (2016). Repetitive mild traumatic brain injury with impact acceleration in the mouse: multifocal axonopathy, neuroinflammation, and neurodegeneration in the visual system. Exp. Neurol. 275, 436–449. 10.1016/j.expneurol.2014.11.004 25450468

[B114] Yttri E. A. Dudman J. T. (2016). Opponent and bidirectional control of movement velocity in the basal ganglia. Nature 533, 402–406. 10.1038/nature17639 27135927 PMC4873380

[B115] Zakaria N. Kallakuri S. Bandaru S. Cavanaugh J. M. (2012). Temporal assessment of traumatic axonal injury in the rat corpus callosum and optic chiasm. Brain Res. 1467, 81–90. 10.1016/j.brainres.2012.05.046 22652307

[B116] Zhang L. Guo Y. Hu H. Wang J. Liu Z. Gao F. (2015). FDG-PET and NeuN-GFAP immunohistochemistry of hippocampus at different phases of the pilocarpine model of temporal lobe epilepsy. Int. J. Med. Sci. 12, 288–294. 10.7150/ijms.10527 25798055 PMC4366634

